# Munc18-1 catalyzes neuronal SNARE assembly by templating SNARE association

**DOI:** 10.7554/eLife.41771

**Published:** 2018-12-12

**Authors:** Junyi Jiao, Mengze He, Sarah A Port, Richard W Baker, Yonggang Xu, Hong Qu, Yujian Xiong, Yukun Wang, Huaizhou Jin, Travis J Eisemann, Frederick M Hughson, Yongli Zhang

**Affiliations:** 1 Department of Cell BiologyYale University School of MedicineNew HavenUnited States; 2 Department of Molecular BiologyPrinceton UniversityPrincetonUnited States; 3 Department of Cellular and Molecular MedicineUniversity of California, San DiegoSan DiegoUnited States; University of Texas Southwestern Medical CenterUnited States; The Barcelona Institute of Science and TechnologySpain

**Keywords:** Sec1/Munc18 (SM) proteins, SNARE proteins, SNARE assembly, optical tweezers, template complex, membrane fusion, *E. coli*

## Abstract

Sec1/Munc18-family (SM) proteins are required for SNARE-mediated membrane fusion, but their mechanism(s) of action remain controversial. Using single-molecule force spectroscopy, we found that the SM protein Munc18-1 catalyzes step-wise zippering of three synaptic SNAREs (syntaxin, VAMP2, and SNAP-25) into a four-helix bundle. Catalysis requires formation of an intermediate template complex in which Munc18-1 juxtaposes the N-terminal regions of the SNARE motifs of syntaxin and VAMP2, while keeping their C-terminal regions separated. SNAP-25 binds the templated SNAREs to induce full SNARE zippering. Munc18-1 mutations modulate the stability of the template complex in a manner consistent with their effects on membrane fusion, indicating that chaperoned SNARE assembly is essential for exocytosis. Two other SM proteins, Munc18-3 and Vps33, similarly chaperone SNARE assembly via a template complex, suggesting that SM protein mechanism is conserved.

## Introduction

Cytosolic Sec1/Munc18 (SM) proteins and membrane-anchored SNARE proteins constitute the core machinery that mediates nearly all intracellular membrane fusion (Rizo and Südhof, 2012; Südhof and Rothman, 2009). In particular, the neuronal SM protein Munc18-1 and its cognate SNAREs syntaxin-1, SNAP-25, and VAMP2 (also called synaptobrevin) drive fusion of synaptic vesicles with the presynaptic plasma membrane (Söllner et al., 1993; Verhage et al., 2000). Fusion releases neurotransmitters into synaptic or neuromuscular junctions, controlling all thoughts and actions. Related SM proteins, Munc18-2 and Munc18-3, are required for cytotoxin release from lymphocytes to kill cancerous or infected cells (Côte et al., 2009) and for glucose uptake (Bryant and Gould, 2011), respectively. Consequently, dysfunctions of SM proteins are associated with neurological and immunological disorders, cancers, diabetes, and other diseases (Bryant and Gould, 2011; Côte et al., 2009; Stamberger et al., 2016).

**Figure 1.**
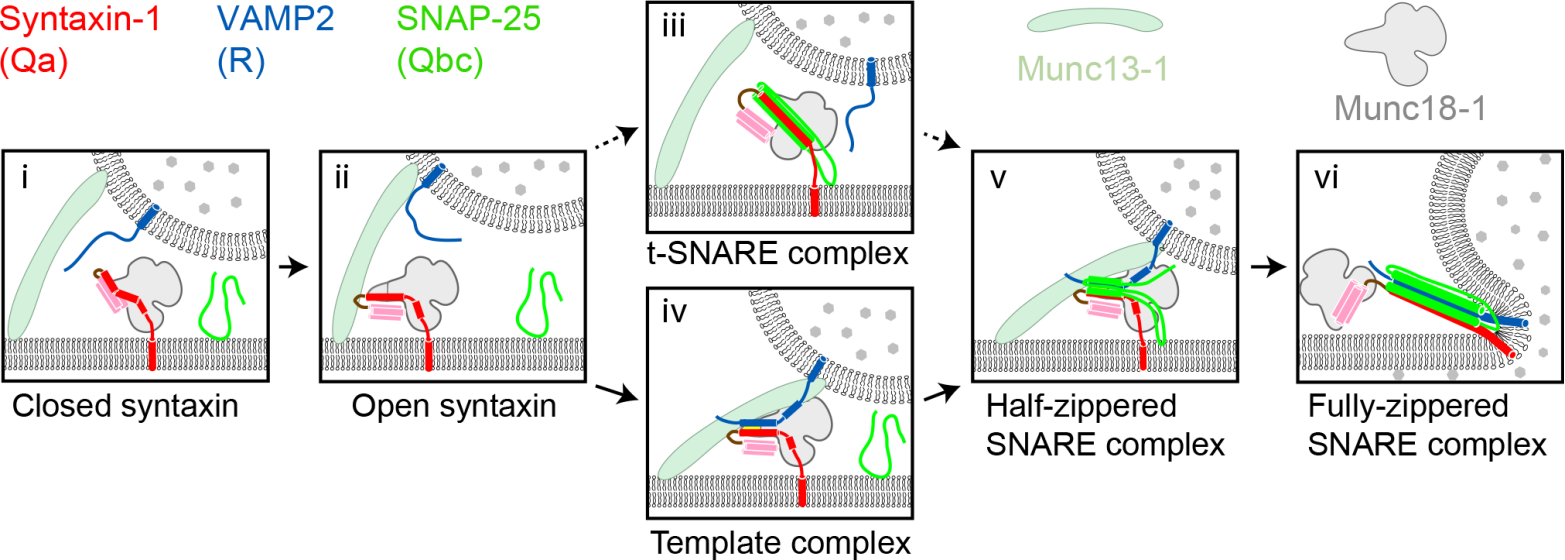
Two potential pathways for Munc18-1-regulated neuronal SNARE assembly. (**i**) Munc18-1 first serves as a syntaxin chaperone and binds syntaxin to inhibit its association with other SNAREs. (**ii**) Closed syntaxin is opened by Munc13-1, a large multifunctional protein that also helps tether vesicles to the plasma membrane and binds, albeit with low affinity, both syntaxin and VAMP2. (**iii**) Open syntaxin may bind SNAP-25 to form a syntaxin:SNAP-25 or a Munc18-1:syntaxin:SNAP-25 complex. (**iv**) Alternatively, open syntaxin may bind VAMP2 to form a Munc18-1:syntaxin:VAMP2 template complex, as proposed here. Both complexes, (**iii**) and (**iv**), have been proposed to be ‘activated’ for SNARE assembly. (**v**) and (**vi**) Other factors such as synaptotagmin (not shown) target the half-zippered SNARE complex to enable calcium-triggered further SNARE zippering and vesicle fusion.

SM proteins regulate the assembly of SNAREs into the membrane-bridging ‘trans-SNARE’ complexes required for membrane fusion (Figure 1) (Baker and Hughson, 2016; Brunger et al., 2018; Gao et al., 2012; Rizo and Südhof, 2012; Shen et al., 2007; Südhof and Rothman, 2009; Sutton et al., 1998). Most SNAREs contain a C-terminal transmembrane anchor, an adjacent SNARE motif, and an N-terminal regulatory domain (NRD). SNARE motifs are 60–70 residues in length, with either glutamine (Q-SNAREs) or arginine (R-SNAREs) residues at a key central position (Fasshauer et al., 1998). SNARE motifs in isolation are intrinsically disordered. By contrast, they are α-helical in fusion-competent SNARE complexes, with three Q-SNARE motifs (designated Qa, Qb, and Qc) and one R-SNARE motif combining to form a parallel four-helix bundle (Sutton et al., 1998). Despite their apparent simplicity, however, the physiological pathway(s) of SNARE assembly have remained enigmatic, as have the specific role(s) of SM proteins (Baker et al., 2015; Jakhanwal et al., 2017; Lai et al., 2017; Ma et al., 2013; Ma et al., 2015; Rizo and Südhof, 2012; Shen et al., 2007; Wickner, 2010; Zhang et al., 2015; Zhou et al., 2013).

SNARE assembly has long been thought to begin with the formation of a t-SNARE complex among the SNAREs – usually Qa, Qb, and Qc – residing on the target membrane (Weber et al., 1998) (Figure 1). According to this view, the neuronal SNAREs syntaxin (Qa-SNARE) and SNAP-25 (Qbc-SNARE, a single protein containing both Qb and Qc SNARE motifs) assemble on the presynaptic plasma membrane, forming a t-SNARE complex that subsequently binds to the synaptic vesicle R-SNARE VAMP2 (Jakhanwal et al., 2017; Pobbati et al., 2006; Shen et al., 2007; Weber et al., 1998; Zhang et al., 2016b). Recent reports have, however, raised doubts about this order of events. In vitro reconstitution experiments suggested that neuronal SNARE assembly begins with a complex between Munc18-1 and syntaxin, requires Munc13-1, and may not involve a syntaxin:SNAP-25 complex (Ma et al., 2013) (Figure 1). Crystal structures of the SM protein Vps33 bound to its cognate Qa- and R-SNARE implied that the SM protein functions as a template, orienting and aligning the two SNARE motifs for further assembly (Baker et al., 2015). Thus, the Qa- and R-SNAREs might be the first to assemble, and only on the surface of an SM template.

Previously, we developed a single-molecule approach based on optical tweezers to dissect SNARE assembly at high spatiotemporal resolution (Gao et al., 2012; Ma et al., 2015; Zhang et al., 2016b; Zorman et al., 2014). Using this method, we measured the folding energy and kinetics of various SNARE complexes. Here, we extend the method to observe SM-mediated SNARE assembly. We detected three template complexes, each of them comprising an SM protein (Munc18-1, Munc18-3, or Vps33) bound to its cognate Qa- and R-SNAREs, and characterized the neuronal template complex in detail using a large panel of mutant proteins. Our results imply that the neuronal template complex is an on-pathway, rate-limiting intermediate in vitro and in vivo. They further suggest that phosphorylation of Munc18-1 can modulate the efficiency of neurotransmitter release by affecting the stability of the template complex. More broadly, our findings imply that membrane fusion in vivo may be controlled by SM proteins through their tunable catalytic activity as SNARE assembly chaperones.

## Results

### Munc18-1, syntaxin, and VAMP2 form a template complex

**Figure 2.**
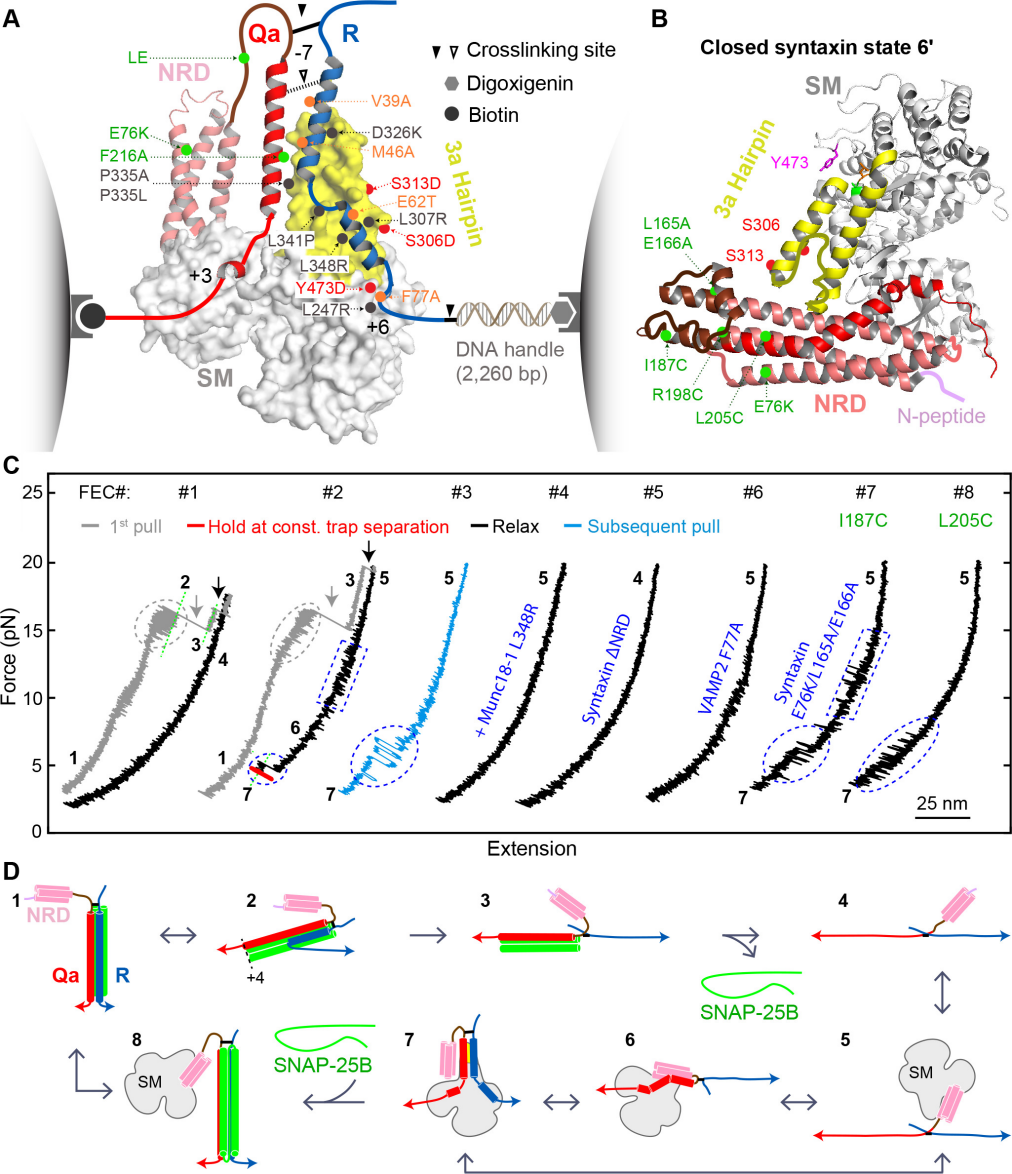
Single-molecule manipulation based on optical tweezers revealed a ternary template complex. (**A**) Experimental setup and structural model of the template complex. Some key mutations tested in this study are indicated by dots: red (phosphomimetic mutations) or gray (others) for Munc18-1, green for syntaxin, and orange for VAMP2. The helical hairpin of Munc18-1 domain 3a is highlighted in yellow. The NRD of syntaxin comprises an N-peptide (a.a. 1–26, see B), a three-helical H_abc_ domain (27–146, deep salmon), and a linker region (147–199, brown). The structural model of the template complex is derived from a similar model of Vps33:Vam3:Nyv1 (Baker et al., 2015) by extending the N-terminal helix of the R-SNARE to the −7 layer, as justified herein. The NRD stabilizes the template complex, but its positioning in this model is arbitrary. See also Figure 2—figure supplement 1. (**B**) Crystal structure of closed syntaxin bound to Munc18-1 (PDB ID 3C98) (Misura et al., 2000). Highlighted are crosslinking sites (I187, R198, and L205), sites of mutations used to destabilize closed syntaxin (E76K, L165A, and E166A, green dots), and sites of phosphomimetic mutations (red dots and Y473). (**C**) Force-extension curves (FECs) obtained in the absence (#1) or presence (other FECs) of Munc18-1 in solution. Throughout the figures, all FECs are color coded in the same fashion: gray for pulling the initial purified SNARE complex, blue for subsequent pulls, black for relaxation, and red for holding the system at constant force. The states associated with different extensions (marked by green dashed lines as needed) are numbered as in Figure 2D. CTD transitions are indicated by gray ovals, NTD unfolding by gray arrows, t-SNARE unfolding by black arrows, syntaxin transitions by blue rectangles, and template complex transitions by blue ovals. See also Figure 2—figure supplement 2 and Figure 2—source datas 1 and 2 in Dataset 1. (**D**) Schematic diagrams of different states: 1, fully assembled SNARE complex; 2, half-zippered SNARE bundle; 3, t-SNARE complex; 4, fully unfolded SNARE motifs; 5, unfolded SNARE motifs with Munc18-1 bound; 6, partially closed syntaxin; 7, template complex; and 8, Munc18-1-bound assembled SNARE complex. In states 2 and 3, the t-SNARE complex is ordered up to the +4 layer (Gao et al., 2012; Ma et al., 2015; Zhang et al., 2016b). The states are numbered according to the same convention throughout the text and figures. 10.7554/eLife.41771.011Figure 2—source data 1.MATLAB figure for the FECs shown in Figure 2C. 10.7554/eLife.41771.012Figure 2—source data 2.Complete time-dependent instantaneous force, extension, and trap separation obtained in a representative single-molecule experiment in the presence of WT 2 µM Munc18-1.Data here correspond to Figure 2C (FECs #2–3) and Figure 2—figure supplement 2.

Previously, we found that the SM protein Vps33 forms binary complexes with the SNARE motifs of Vam3 (Qa-SNARE) and Nyv1 (R-SNARE), as well as a ternary complex containing all three proteins (Baker et al., 2015). Crystal structures of the two binary complexes revealed that the Qa-SNARE and the R-SNARE bind to adjacent sites on the SM protein and led to a model of the template complex in which the two SNARE motifs are ‘half-zippered’. An analogous template complex might form during the assembly of the neurotransmitter release machinery (Sitarska et al., 2017), but direct evidence is lacking. To investigate further, we used an optical tweezers-based strategy to directly observe neuronal SNARE assembly and disassembly in the presence of Munc18-1. To mimic a trans-SNARE complex, pre-assembled SNAREs were attached via the C termini of the Qa- and R-SNARE motifs to beads (Gao et al., 2012). The same two SNARE motifs were covalently linked near their N termini through an engineered disulfide bond to form a Qa-R-SNARE conjugate (Figure 2A and Figure 2—figure supplement 1). This tactic permitted us to conduct repeated rounds of force-induced unfolding/disassembly (‘pulling’) and potential refolding/assembly (‘relaxation’) in a single experiment.

**Figure 2—figure supplement 1.**
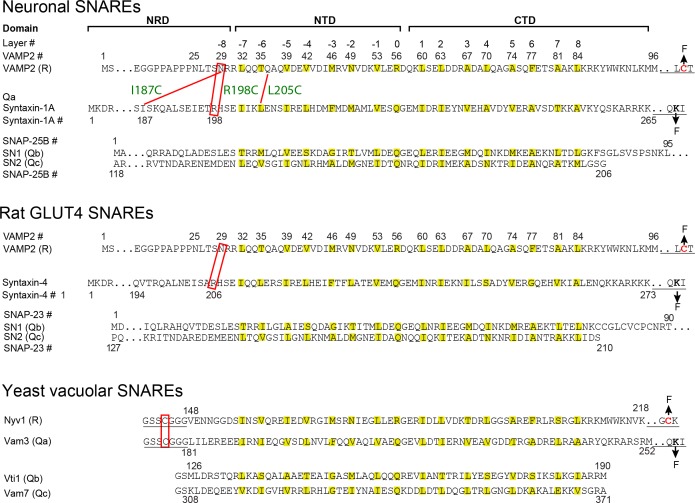
Sequences, domains, and crosslinking sites of the SNARE proteins used in this study. The amino acids in hydrophobic layers (from −7 to +8) and the central ionic layer (0 layer) are colored yellow. The underlined sequences are added to facilitate crosslinking of Qa and R SNAREs, crosslinking of R SNAREs to DNA handles, and attachment of Qa SNAREs to bead surfaces (Figure 2A). The pulling sites are indicated by arrows labeled by F (for Force). The crosslinking sites are indicated by red rectangles or red lines. For neuronal SNAREs, the three crosslinking sites tested are designated by their corresponding syntaxin residues (R198C, I187C, and L205C, Figure 2B,C). N-terminal crosslinking facilitated formation of the template complex by destabilizing the closed syntaxin, increasing the local SNARE concentrations, and minimizing nonspecific Munc18-1-VAMP2 and SNARE-SNARE interactions (Brunger, 2005; Lai et al., 2017; Sitarska et al., 2017); see the Materials and methods section ‘Estimation of the affinity between VAMP2 and Munc18-bound syntaxin in the absence of crosslinking’ for additional discussion. To facilitate assembly of the Qa-R conjugate and the SNARE-DNA tether, we typically crosslinked the Qa- and R-SNAREs in purified SNARE complexes and then removed the Qbc-SNAREs by unfolding the complexes. The force-extension curves (FECs) corresponding to the first pull to unfold these pre-assembled SNARE complexes are shown in gray throughout the text.

**Figure 2—figure supplement 2.**
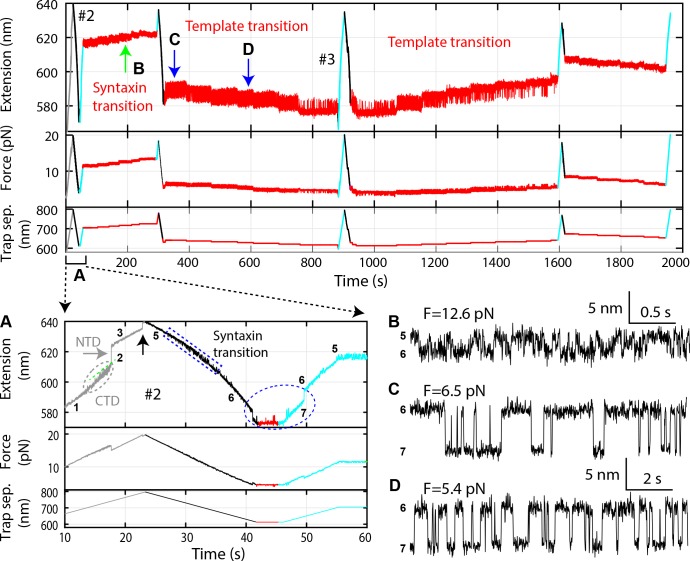
Time-dependent extension (top panel), force (middle panel), and trap separation (bottom panel) for a typical experiment to test template complex formation (Figure 2—source data 2). Data here and FECs #2 and #3 in Figure 2C are acquired on the same Qa-R SNARE conjugate, with the same pulling round numbering. Close-up views of different time regions indicated by A-D are shown: (**A**) Close-up view of the first round of pulling and relaxation. Regions of characteristic transitions are indicated: the CTD transition by the gray oval, the NTD unfolding by the gray arrow, the t-SNARE complex unfolding and SNAP-25B dissociation by the black arrow, the syntaxin open-partial closing transition by the blue rectangle, and the template complex transition by the blue oval. States associated with different extensions are indicated by the corresponding state numbers (Figure 2D). Note that binding of Munc18-1 to the NRD in state five did not cause any change in the extension of the Qa-R conjugate compared to the state 4, thus could not directly be detected by our assay. (**B**) The extension-time trajectory demonstrates reversible partial closing of syntaxin at a constant mean force of 12.6 pN. (**C–D**) Extension-time trajectories at two constant trap separations or mean forces showing reversible folding and unfolding of the template complex. Throughout the figures, all FECs and time-dependent trajectories were mean-filtered with a time window of 10 ms, except for the extension-time trajectories with syntaxin opening-partial closing transitions, which were filtered with a time window of 3 ms.

**Figure 2—figure supplement 3.**
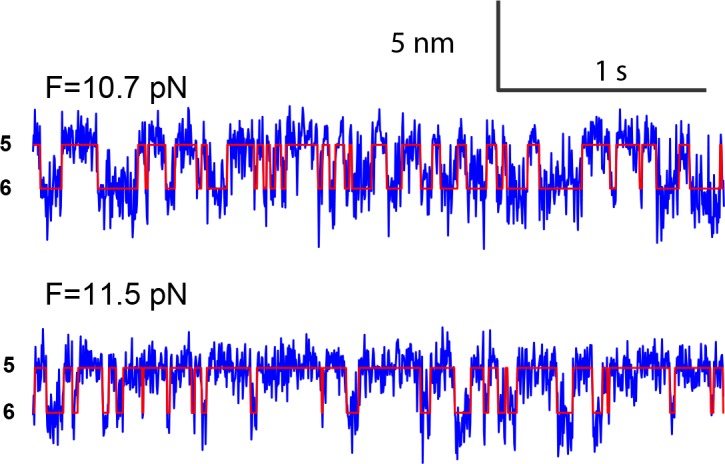
Extension-time trajectories at two constant mean forces showing the opening-closing transition of the partially closed syntaxin molecule.

**Figure 2—figure supplement 4.**
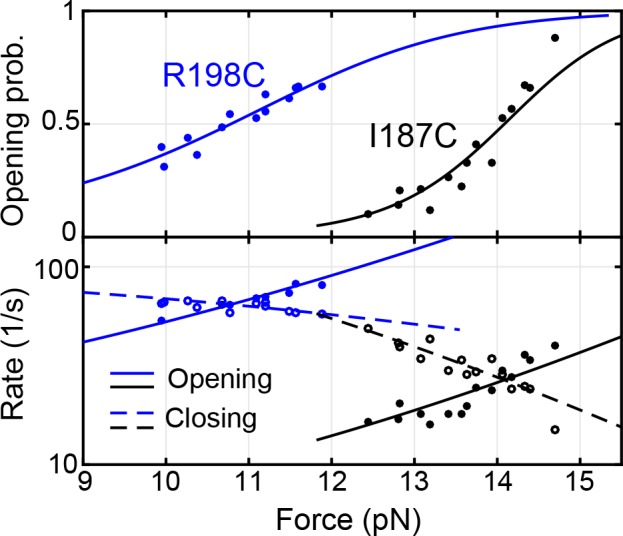
Force-dependent syntaxin opening probabilities (top panel) and opening and closing rates (bottom panel) obtained by pulling syntaxin from the two N-terminal sites, R198C and I187C (Figure 2—figure supplement 1). Curves are best model fits to derive the energies and kinetics at zero force associated with the transitions, with solid curves for unfolding and dashed curves for folding. The unfolding energies of the closed syntaxin (7.2 ± 0.2 k_B_T) and of the partially closed syntaxin (2.6 ± 0.2 k_B_T) are much smaller than the dissociation energy between syntaxin and Munc18-1 previously measured based on a two-state binding and unbinding process (~22 k_B_T) (Burkhardt et al., 2008; Sitarska et al., 2017). Our data reveal an intermediate state for the association and dissociation process, in which syntaxin is open, but Munc18-1 remains bound to syntaxin, likely to the NRD (Ma et al., 2015) (Figure 2D, state 5).

**Figure 2—figure supplement 5.**
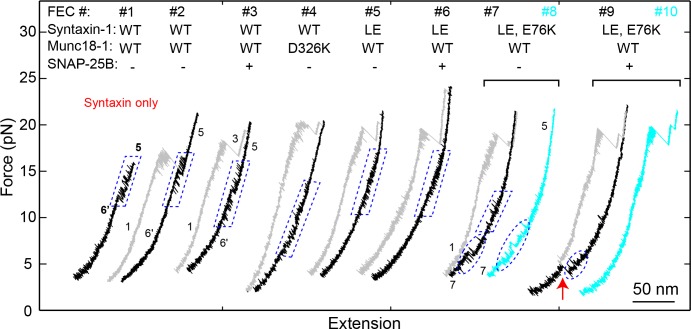
FECs of Qa only (#1) or the Qa-R SNARE conjugate (other FECs) pulled from Site I187C in the presence of 2 μM Munc18-1 without (-) or with (+) 60 nM SNAP-25B. The wide-type (‘WT’) syntaxin-1 here denotes syntaxin-1A (a.a. 1–265, I187C, C145S; see ‘SNARE protein constructs’), with additional mutations indicated. FECs in each bracket were obtained on the same Qa-R conjugate. Different transitions are marked: blue dashed parallelograms for syntaxin opening-closing transitions, blue dashed ovals for template complex transitions, and the red arrow for SNAP-25B binding and full SNARE assembly. To pull syntaxin alone, we directly crosslinked the DNA handle to syntaxin I187C in the absence of VAMP2. The resultant FEC (#1) revealed the same high-force transition as those obtained by pulling the Qa-R conjugate crosslinked at I187C, indicating that the high-force transition indeed resulted from transition of syntaxin alone (compare to the syntaxin transition in #2–3). The WT syntaxin was fully closed by Munc18-1, which inhibited formation of the template complex (#2–3) in the absence (#2) and presence (#3) of SNAP-25B. Munc18-1 D326 stabilizes a furled loop at the tip of the 3a helical hairpin in the closed syntaxin state (Figure 2B). The mutation D326K helps unfurl the loop (Sitarska et al., 2017), which abolished the partially closed syntaxin and facilitated formation of the template complex when the Qa- and R-SNAREs were crosslinked at Site R198C (Figure 4A; Table 1). At Site I187C, D326K destabilized the closed syntaxin, but was not sufficient to open the closed syntaxin for template complex formation (#4). The LE mutation (L165A/E166A) decreased the extension change associated with the syntaxin transition (compare #5 to #1–3), indicating that the LE mutation destabilized the closed syntaxin conformation (Burkhardt et al., 2008). Nevertheless, neither template complex formation (#5 and #6) nor Munc18-1-chaperoned SNARE assembly (#6) was observed, suggesting that syntaxin with the LE mutation is still largely closed, consistent with recent results (Colbert et al., 2013). Therefore, we made an additional mutation E76K that was expected to further weaken the closed syntaxin conformation (Figure 2B). Indeed, the combined mutation partially opened the syntaxin conformation and promoted formation of the template complex, as seen from the small high force transition (#7) and appearance of the low force transition (#7 and #8), respectively. Correspondingly, Munc18-1-chaperoned SNARE assembly was observed in the presence of SNAP-25B (red arrow) in a manner that depended on the template complex (#9 and #10). Under this condition, the probabilities of detecting the partially closed syntaxin and the template complex were 0.66 and 0.39, respectively, and the probability of detecting SNARE assembly after template complex formation was 0.48.

**Figure 2—figure supplement 6.**
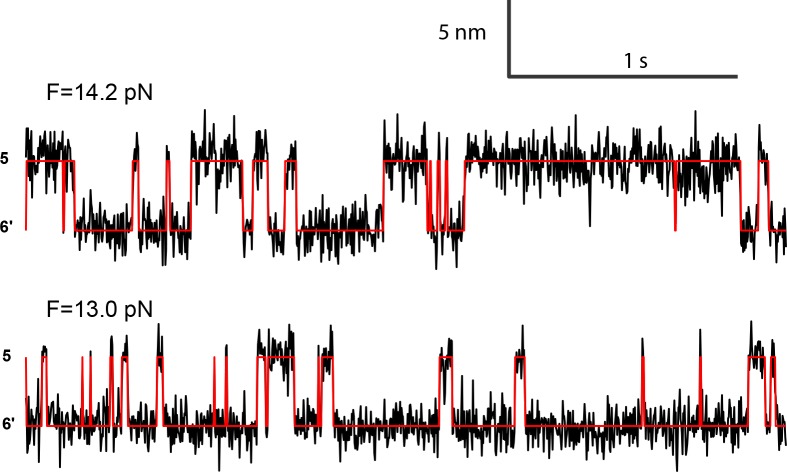
Extension-time trajectories at two constant mean forces showing the opening-closing transition of the syntaxin molecule pulled from the crosslinking site I187C (Figure 2—figure supplement 1). The red curves are idealized state transitions derived from hidden-Markov modeling. State 6' represents the fully closed syntaxin (Figure 2B).

**Figure 2—figure supplement 7.**
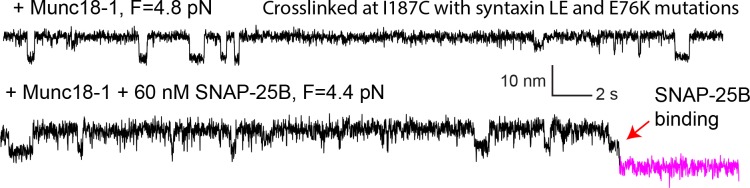
Extension-time trajectories showing conformational transitions of the template complex transition pulled from Site I187C in the absence (top trace) and presence (bottom) of 60 nM SNAP-25B. The unfolding energy of the template complex crosslinked at I187C is estimated to be 4.8 ± 0.3 k_B_T, close to the unfolding energy of 5.2 ± 0.1 k_B_T of the template complex crosslinked at R198C. Furthermore, in the presence of SNAP-25B, the template complex facilitates SNAP-25B binding and SNARE assembly (red arrow).

Munc18-1 binds both the Qa-SNARE syntaxin (with nanomolar affinity) and the R-SNARE VAMP2 (with micromolar affinity) (Burkhardt et al., 2008; Misura et al., 2000; Parisotto et al., 2014; Sitarska et al., 2017). Formation of a ternary template complex has not, however, been reported. This is presumably because Munc18-1 and syntaxin, in their high-affinity complex, both adopt conformations that preclude VAMP2 binding (Baker et al., 2015; Misura et al., 2000; Sitarska et al., 2017) (Figure 2B). In particular, the SNARE motif and NRD of syntaxin interact to create an autoinhibited or ‘closed’ conformation (Misura et al., 2000). Opening syntaxin, and thereby permitting SNARE assembly, requires Munc13-1 (Ma et al., 2011; Ma et al., 2013; Wang et al., 2017; Yang et al., 2015). To bypass the requirement for Munc13-1 in our single-molecule experiments, we attempted to destabilize the closed conformation of syntaxin without abolishing its interactions with Munc18-1 or the other SNAREs. Among the strategies we evaluated, the simplest was to form the Qa-R-SNARE conjugate by crosslinking syntaxin R198C and VAMP2 N29C (Figure 2A, solid arrowhead; Figure 2—figure supplement 1). In closed syntaxin, residue 198 is buried against the NRD (Figure 2B). As shown below, involving this residue in a disulfide bond destabilized and partially opened Munc18-bound syntaxin, presumably via localized unfolding.

We began by pulling the fully folded neuronal SNARE complex, containing crosslinked syntaxin and VAMP2 as well as SNAP-25B, in the absence of Munc18-1. The resulting force-extension curve (FEC) revealed that, as expected based on our previous work (Gao et al., 2012; Ma et al., 2015), the SNARE complex disassembled in at least three steps (Figure 2C, FEC #1, gray curve). These force-induced disassembly steps are schematically depicted in Video 1 and in Figure 2D as transitions from states 1↔2→3→4. 1↔2 represents reversible unfolding of the C-terminal half of the VAMP2 SNARE motif (CTD; Figure 2C, gray oval), 2→3 represents irreversible unfolding of the N-terminal half of the VAMP2 SNARE motif (NTD; gray arrow), and 3→4 represents irreversible unfolding of the syntaxin SNARE motif (black arrow). 3→4 was accompanied by release of SNAP-25B. Relaxing the resulting Qa-R-SNARE conjugate revealed a featureless FEC, as expected for an unfolded polypeptide (Figure 2C, FEC #1, black curve) (Gao et al., 2012; Ma et al., 2015).

**Video 1.** SNARE complex unfolding and subsequent template complex formation as inferred from single-molecule measurements. The proposed state transitions associated with FEC #2 in Figure 2C or Figure 2—figure supplement 2 are simulated.

We next asked whether our single-molecule assay could be used to detect and characterize the predicted template complex (Figure 2A). The addition of 2 μM Munc18-1 had little effect on the unfolding pathway of the initial syntaxin/VAMP2/SNAP-25B complex (Figure 2C, compare gray curves in FECs #1 and #2; Video 1). However, the presence of Munc18-1 had a striking effect on the FEC of the remaining Qa-R-SNARE conjugate. Specifically, relaxing (Figure 2C, #2, black trace) and then pulling (Figure 2C, #3, blue trace) the Qa-R-SNARE conjugate revealed two Munc18-1-dependent features (Figure 2—figure supplement 2). In about 40% of the FECs, we observed a small flickering signal at 10–15 pN (Figure 2C, #2, blue rectangle; Figure 2—figure supplement 3). We attribute this transition (5↔6) to the reversible folding/unfolding of the partially closed syntaxin conformation induced by Munc18-1 (state 6 in Figure 2D). More importantly, in about 50% of the FECs, we observed prominent flickering signals at 3–7 pN (Figure 2C, #2–3, blue ovals). In a given FEC, these transitions could be sequential (#2, 5↔6 followed by 6↔7) or cooperative (#3, 5↔7). As described in detail below, extensive evidence supports the conclusion that this transition (6↔7 or 5↔7) results from the reversible, cooperative formation and unfolding of the predicted template complex (Figure 2A; state 7 in Figure 2D). For example, the probability of observing this transition was greatly reduced when either the Munc18-1:VAMP2 interaction or the Munc18-1:syntaxin interaction was abrogated (Burkhardt et al., 2008; Parisotto et al., 2014) (Figure 2C, #4–5; Table 1).

**Table 1.** Properties of the neuronal template complex.

SNARE or SM	Mutation or truncation	Unfolding energy (k_B_T)	Equilibrium force^*^ (pN)	Folding rate (s^−1^)	Unfolding rate (s^−1^)	Partially closed syntaxin^†^	Template formation			SNAP-25 binding	
						Prob.	Prob.^‡^	N^§^		Prob.^¶^	N^**^
**WT**	-	5.2 (0.1)	5.1 (0.1)	132	0.7	0.4	0.5	346		0.7	50
**Munc18-1**	L247R	1.6 (0.3)	2.3 (0.1)	-	-	0.3	0.3	99		0.7	6
	T248G	2.9 (0.2)	3.1 (0.1)	-	-	0	0.3	155		0.3	16
	L247A/ T248G	<1.5^***^	-	-	-	0	0	241		-	-
	S306D^¶¶^	5.8 (0.1)	5.6 (0.1)	184	0.6	0.4	0.9	123		0.9	53
	L307R	4.1 (0.2)	4.6 (0.1)			0.07	0.43	114		0.58	19
	S313D^¶¶^	6.1 (0.2)	5.7 (0.1)	568	1.5	0.4	1	162		0.8	70
	Δ324– 339^††,‡‡^	<1.5^***^		-	-	0	0	105		0	0
	D326K^¶¶^	6.5 (0.2)	5.7 (0.1)	420	0.6	0.03	0.9	103		1	27
	P335A^§§^	6.0 (0.3)	5.9 (0.1)	258	0.5	0.02	0.7	155		0.9	11
	P335L^§§^	4.3 (0.1)	4.8 (0.1)	17	0.2	0.4	0.3	224		0.8	36
	L341P^§§^	<1.5^***^		-	-	0.06	0.04	176		0.5	4
	L348R^††,‡‡^	<1.5^***^		-	-	0.02	0.04	222		0.7	6
	Y473D^‡‡^	4.0 (0.1)	4.3 (0.2)	-	-	0	0.1	395		0.5	24
**VAMP2**	L32G/Q33G	3.4 (0.2)	3.9 (0.1)	310	10	0.4	0.6	170		0.06	33
	V39D	3.8 (0.4)	3.9 (0.2)	90	2	0.3	0.1	175		0.8	13
	M46A	5.2 (0.4)	5.1 (0.2)	130	0.7	0.3	0.5	52		0.8	13
	E62T^††^	4.1 (0.2)	4.8 (0.2)	107	5	0.4	0.5	104		0.4	23
	S61D/ E62T^††^	3.6 (0.2)	4.1 (0.1)			0.4	0.7	56		0.2	12
	Q76A^††^	4.7 (0.2)	4.8 (0.1)	166	2	0.4	0.6	62		0.3	12
	F77A^‡‡^	1.5 (0.3)	2.3	-	-	0.5	0.1	121		0.5	6
	A81G/A82G	5.0 (0.3)	4.9 (0.2)	130	0.8	0.4	0.5	149		0.4	42
	Δ85–94	5.1 (0.2)	5.0 (0.1)	120	0.7	0.4	0.5	87		0.7	29
**Syntaxin-1**	ΔNRD^††,‡‡^	<1.5^***^	-	-	-	0	0.08	105		0.2	12
	ΔN- peptide^††,‡‡^	3.2 (0.2)	4.6 (0.1)	42	2	0.03	0.5	328		0.4	46
	ΔH_abc^‡‡^_	<1.5^***^	-	-	-	0	0.06	140		0.5	4
	L165A/E166A (LE)^¶¶^	6.7 (0.2)	6.1 (0.1)	406	0.5	0.07	0.7	83		0.9	26
	LE/E76K	6.4 (0.2)	6.0 (0.2)	123	0.2	0.07	0.9	81		0.7	30
	I202G/I203G	3.0 (0.3)	3.8 (0.1)	240	12	0.4	0.5	177		0.4	33
	F216A	3.7 (0.1)	5.1 (0.1)	82	2	0	0.6	155		0.9	32
	I230G/D231G/ R232G^†††^	3.6 (0.2)	4.3 (0.1)	-	-	0	0.5	111		0.4	7
	I233G/E234G/ Y235G^†††^	3.0 (0.2)	4.1 (0.1)	-	-	0	0.6	122		0.7	30
	V237G/E238G/ H239G	5.2 (0.2)	4.9 (0.1)	124	0.7	0.01	0.3	182		0.4	14
	T251G/K252G	5.2 (0.1)	4.9 (0.1)	126	0.7	0.5	0.8	197		0.7	47
	Δ255–264	5.4 (0.2)	5.1 (0.1)	140	0.6	0.5	0.5	134		0.7	29
Syntaxin-1	L165A/E166A	6.6 (0.2)	6.2 (0.1)	72	0.1	0.2	0.9	85		0.2	11
Munc18-1	D326K^¶¶^										

^*^ Mean of two average forces for the unfolded and folded states when the two states are equally populated (Rebane et al., 2016). The equilibrium force of the template complex generally correlates with its unfolding energy. The number in parentheses is the standard error of the mean. ^† ^Detected as the syntaxin- and Munc18-1-dependent transition in the force range of 10–15 pN.

^‡^ Probability per relaxation or pulling measured in the absence of SNAP-25B. ^§^ Total number of pulling or relaxation FECs acquired, in which transitions of the template complex or syntaxin are scored, including their average equilibrium forces and extension changes.

^¶^ Probability of SNAP-25B binding and SNARE assembly per relaxation upon formation of the template complex. ^**^ Total number of relaxation FECs containing the template complex transition.

^††^ Mutation that reduces membrane fusion in vitro (Parisotto et al., 2014; Shen et al., 2010; Shen et al., 2007). ^‡‡^ Mutation that impairs exocytosis or neurotransmitter release in vivo (Meijer et al., 2018; Munch et al., 2016; Walter et al., 2010).

^§§^ Mutation associated with epilepsy (Stamberger et al., 2016). ^¶¶^ Mutation that *enhances* membrane fusion in vitro or neurotransmitter release in the cell (Genc et al., 2014; Gerber et al., 2008; Lai et al., 2017; Munch et al., 2016; Parisotto et al., 2014; Richmond et al., 2001).

*** Unfolding energy below the detection limit of our method, estimated to be 1.5 k_B_T, or not available due to no, infrequent, or heterogeneous template complex transition. ^†††^ In the observed template complex transition, the template complex frequently dwelled in the unfolded state for an unusually long time (Figure 3—figure supplement 1). Thus, the transition is no longer two-state.

### Stability and conformation of the template complex

**Figure 3.**
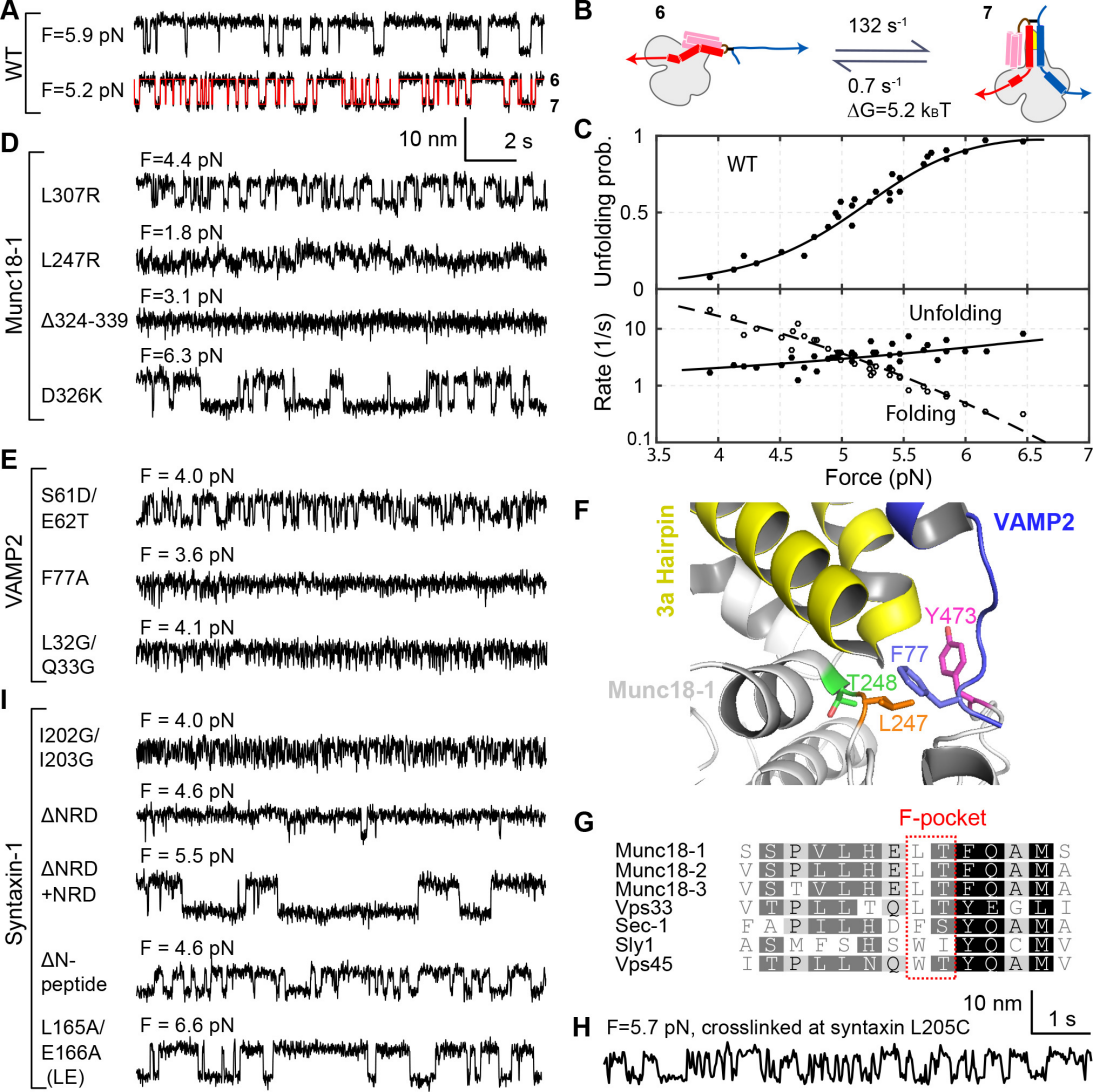
Stability, conformation, and folding kinetics of the template complex. (**A, D, E, I**) Extension-time trajectories at constant mean forces with the WT template complex (**A**) or its variants containing indicated mutations in Munc18-1 (**D**), VAMP2 (**E**), or syntaxin (**I**). The red trace in A shows an exemplary idealized trajectory derived from hidden Markov modeling. Trajectories in A, D, E, and I share the same scale bars. See also Figure 3—figure supplement 1 and Figure 3—source datas 1 and 2 in Dataset 1. (**B**) Diagram illustrating the transition between the partially closed syntaxin state (state 6 in Figure 2D) and the template complex state (state 7); rates and energies are derived from panel C. (**C**) Force-dependent unfolding probabilities (top) and transition rates (bottom). Best model fits (solid and dashed curves) reveal the stability and folding and unfolding rates of the template complex at zero force (Figure 4, Table 1, and Figure 3—source data 3 in Dataset 1). (**F**) Structural model of VAMP2 F77 anchored in the F-pocket in Munc18-1 composed of L247 and T248, which is covered by Y473. The model was derived by superimposing the structures of Munc18-1:syntaxin (Figure 2B; 3C98) and Vps33:Nyv1 (5BV0). (**G**) Sequence alignment showing F-pocket sequence conservation among SM proteins. (**H**) Extension-time trajectory of the WT template complex at 5.7 pN. The Qa-R SNAREs were crosslinked between syntaxin L205C and VAMP2 Q36C (Figure 2A, open arrowhead). See also Figure 2—figure supplement 1 and Figure 3—source data 2 in Dataset 1. 10.7554/eLife.41771.019Figure 3—source data 1.MATLAB figure corresponding to Figure 3A with an additional trace at force F = 5.0 pN. 10.7554/eLife.41771.020Figure 3—source data 2.MATLAB figure containing expanded traces shown in Figure 3D,E,I,H. 10.7554/eLife.41771.021Figure 3—source data 3.MATLAB figure for Figure 3C.

To examine the stability and folding/unfolding kinetics of the template complex, we monitored the 6↔7 transition over a range of constant mean forces (Figure 3A–C; Video 1). Detailed analyses of the extension trajectories (Figure 3A) revealed the force-dependent unfolding probability and transition rate of the template complex (Figure 3C). Specifically, the midpoint of the template complex folding/unfolding transition occurs at 5.1 ± 0.1 pN (mean ± SEM throughout the text) with an associated extension change of 5.42 ± 0.08 nm. Extrapolating the force-dependent measurements to zero force using a nonlinear model (Gao et al., 2012; Rebane et al., 2016; Zhang et al., 2016a) (see also Materials and methods), we obtained the unfolding energy (5.2 ± 0.1 k_B_T or 3.1 ± 0.1 kcal/mol) and lifetime (1.4 s) of the template complex (Figure 3B). Comparable analysis of the folding/unfolding of the partially closed syntaxin (5↔6) allowed us to estimate its unfolding energy as well (2.6 ± 0.2 k_B_T; Figure 2—figure supplement 4). The extension changes associated with these transitions (5→6→7) are consistent with a structural model of the template complex based on the crystal structures of Vps33:Nyv1 and Vps33:Vam3 (Baker et al., 2015) coupled with a worm-like chain model for the unfolded regions of the SNARE proteins and the DNA handle (Marko and Siggia, 1995; Rebane et al., 2016) (Figure 2A). Importantly, the same template complex was observed when we used an alternative Qa-R-SNARE crosslinking site at syntaxin I187C and VAMP2 N29C, but only in conjunction with additional NRD mutations E76K, L165A, and E166A to destabilize the closed conformation of syntaxin (Figure 2C, #7; Figure 2B; Figure 2—figure supplements 5–7). Thus, observation of the template complex was independent of the crosslinking site, requiring only that the closed conformation be destabilized (Hu et al., 2011) (see further analysis in the section ‘Estimation of the affinity between VAMP2 and Munc18-bound syntaxin in the absence of crosslinking’ in Materials and methods).

**Figure 3—figure supplement 1.**
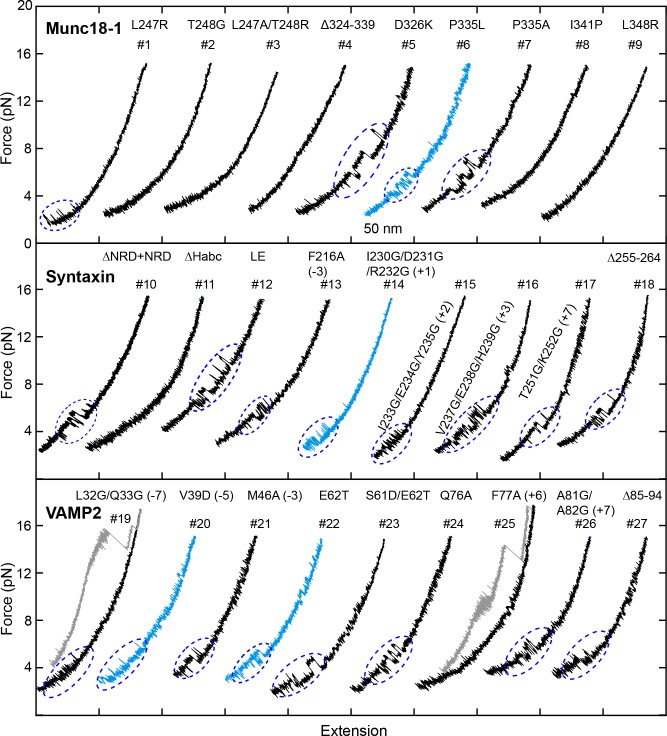
FECs obtained in the presence of 2 µM Munc18-1. Dashed blue ovals mark template complex transitions. Note that the partially closed syntaxin state was abrogated by modifications that are known to destabilize the closed syntaxin, including syntaxin ΔNRD (Burkhardt et al., 2008), the LE mutation (Dulubova et al., 1999; Ma et al., 2011), and Munc18-1 Δ324–339, P335L, and D326K (Munch et al., 2016; Parisotto et al., 2014; Sitarska et al., 2017) (Table 1). For syntaxin LE mutation and I230G/D231G/R232G and Munc18-1 P335A and D326K, template complexes generally formed directly from the open syntaxin (Table 1).

**Figure 3—figure supplement 2.**
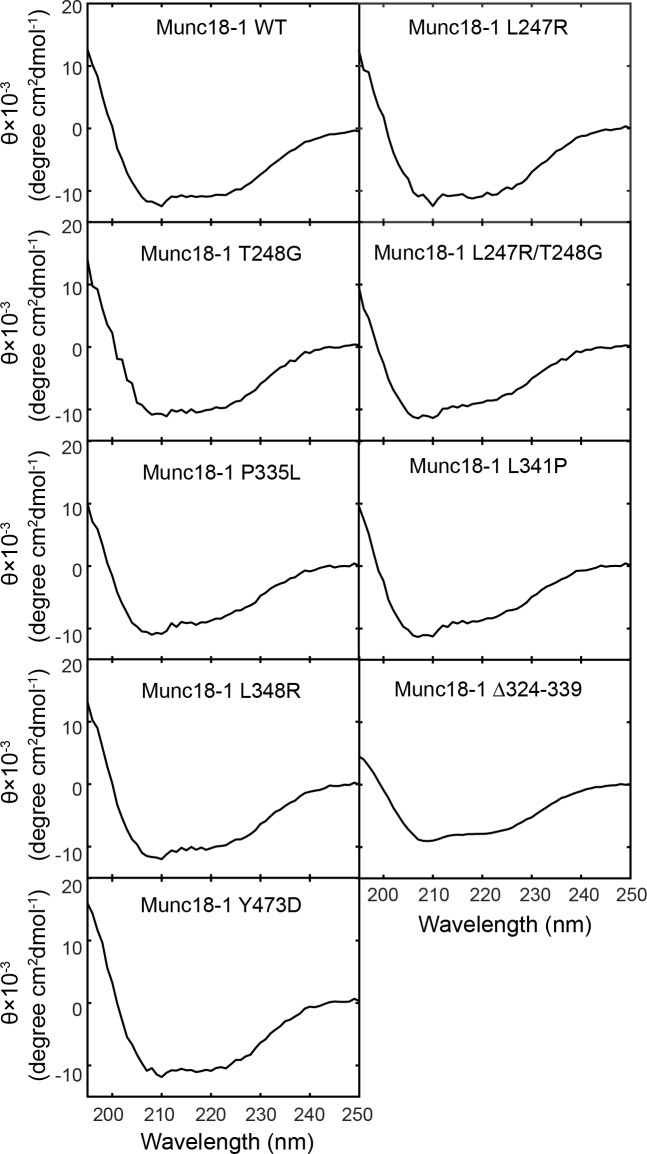
Circular Dichroism (CD) spectra show that the mutations tested in our experiments do not significantly alter Munc18-1 folding. The CD spectra of Munc18-1 mutants that abolished or weakened the template complex are shown, including Munc18-1 F-pocket mutations L247R, T248G, L247A/T248G, disease-related mutations L341P and P335L, phosphomimetic mutation Y473D, and L348R (Parisotto et al., 2014). All the mutant proteins displayed CD spectra closely resembling that of wild-type Munc18-1. In addition, no aggregation at concentrations below 5 mg/ml was noted during the purification of these mutant proteins. These observations suggest that the overall folding of the Munc18-1 proteins are not altered by the mutations. Our results do not however rule out the possibility that some mutations may destabilize the folded protein.

**Figure 3—figure supplement 3.**
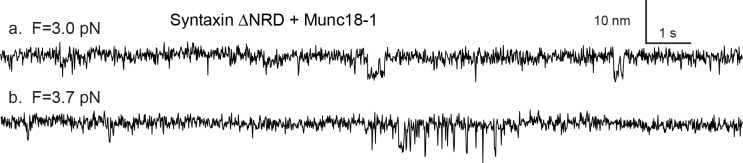
Snapshots of the extension-time trajectories at constant mean forces showing sporadic folding of the template complex in the absence of syntaxin NRD.

**Figure 4.**
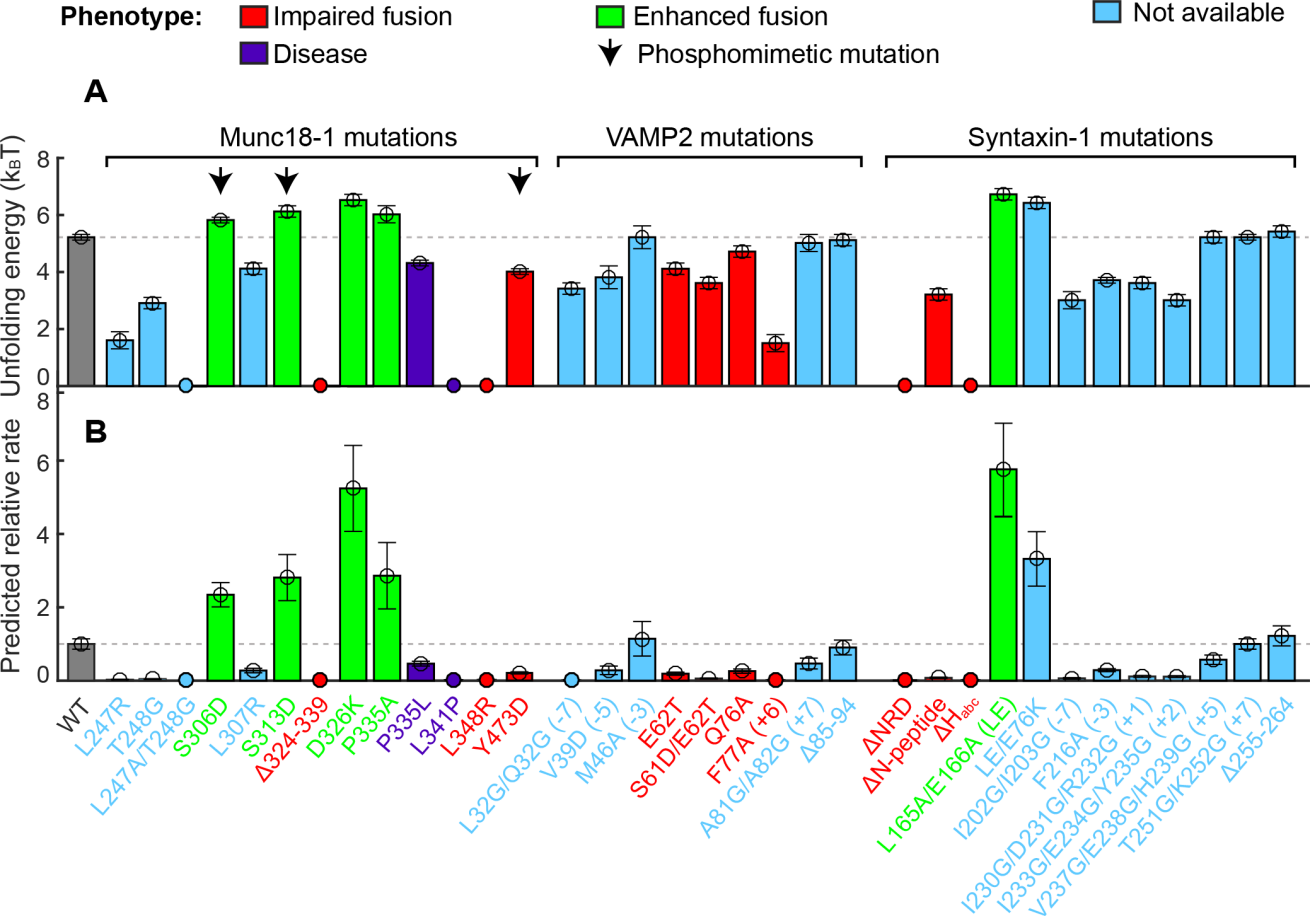
Stability of the template complex correlates with SNARE-mediated membrane fusion and neurotransmitter release. (**A**) Unfolding energy of the WT and mutant template complexes; see also Table 1. Unfolding energy that is less than our detection limit (1.5 k_B_T) is plotted as zero. The unfolding energy is derived from the work required to reversibly unfold the template complex (Rebane et al., 2016). The work is measured as the equilibrium force multiplied by the extension change associated with the template complex transition; see also Figure 3C and Figure 3—figure supplement 1. Numbers in parentheses after SNARE mutant names indicate the layer numbers associated with the corresponding mutations. Error bars indicate standard errors of the mean. See also Figure 4—source data 1. (**B**) Relative rate of SNARE assembly and membrane fusion calculated using Equation 1, derived under the assumption that the rate is determined by the stability of the template complex. 10.7554/eLife.41771.023Figure 4—source data 1.Data summary table for the results shown in Figure 4.

We used a battery of mutant proteins to test our structural model of the template complex in greater detail (Figure 2A). A salient feature of the model is the pivotal role played by a pair of α-helices (a.a. 298–359) within domain 3a of Munc18-1 (Baker et al., 2015; Sitarska et al., 2017) (yellow in Figure 2A,B). These α-helices form an extended helical hairpin that interacts extensively with the NTD of syntaxin and with both the NTD and the CTD of VAMP2 (Figure 2A and Figure 2—figure supplement 1). Many domain 3a mutations within (L307R, P335L, L341P, L348R) or adjacent (L247R, T248G) to the helical hairpin destabilized the template complex (Figures 3D and 4A, and Figure 3—figure supplement 1; Table 1 and references therein). An internal deletion that removes the distal portion of the helical hairpin (Munc18-1 Δ324–339) abolished formation of the template complex altogether. Notably, two helical hairpin mutations – D326K and P335A – actually stabilized the template complex; both of these mutations are associated with enhanced Munc18-1 function in vitro and in vivo (Munch et al., 2016; Parisotto et al., 2014; Sitarska et al., 2017). Three phosphomimetic mutations (S306D, S313D, and Y347D) are discussed later. As judged by circular dichroism spectropolarimetry, none of the mutations we tested had a significant effect on the overall structure of Munc18-1 (Figure 3—figure supplement 2). Overall, the consequences of Munc18-1 mutations are consistent with our structural model.

Reciprocally, we investigated the impact of SNARE motif mutations that appeared likely to affect the SNARE:Munc18-1 interface. Although VAMP2 M46A did not have a significant effect, the rest (VAMP2 S61D/E62T, E62T, Q76A, and F77A; syntaxin F216A, I230G/D231/R232G, and I233G/E234G/Y235G) all destabilized the template complex (Figure 3E and 4A; Table 1; Figure 3—figure supplement 1). The VAMP2 residue Phe 77, located at the so-called +6 layer (Figure 2—figure supplement 1), appears to play an especially important role. In our model of the template complex, the side chain of Phe 77 inserts into a deep, hydrophobic pocket in domain 3a, with Leu 247 and Thr 248 residues at the bottom (Figure 3F). Phe 77 is highly conserved among R-SNAREs, whereas Leu 247 and Thr 248 are highly conserved among SM proteins (Figure 3G). Substituting Phe 77 with Ala dramatically reduced the formation probability of the template complex to 0.06 and its unfolding energy to 1.5 ± 0.3 k_B_T, the lower limit of our assay (Figure 2C, #6; Figure 3E; Figure 3—figure supplement 1). Similarly, Munc18-1 mutations in the hydrophobic pocket strongly impaired (for L247R or T248G) or totally abolished (for L247A and T248G together) formation of the template complex (Figure 3D; Figure 4A; Table 1). Taken together, our mutagenesis results confirm that the stability of the template complex depends on extensive interactions between Munc18-1 and the two SNARE motifs, including a key anchoring role for the +6 layer Phe of VAMP2.

In the binary Vps33:SNARE crystal structures we reported previously (Baker et al., 2015), only the central regions of each SNARE motif (Qa-SNARE layers −4 to +3; R-SNARE layers −4 to +6) contact the SM template, whereas both ends of each SNARE motif are likely disordered. In the ternary template complex, however, the two SNARE motifs may be correctly zippered all the way to their N-termini. First, −7 layer mutations (VAMP2 L32G/L33G or syntaxin I202G/I203G) destabilized the template complex, as did a −5 layer mutation (VAMP2 V39D) (Figure 3E,I; Figure 4; Table 1). Second, crosslinking the SNAREs at the −6 layer (via syntaxin L205C and VAMP2 Q36C; open arrowhead in Figure 2A; Figure 2—figure supplement 1) (Ma et al., 2015) enhanced the probability of observing the template complex to 0.93 (Figure 2C, #8; Figure 3H). Taken together, these data suggest that the −6 layer is properly aligned in the template complex and that the N-terminal regions from layers −7 to −5 – which are unlikely to contact Munc18-1 but nevertheless contribute to the stability of the complex – are correctly zippered (Figure 2A). By contrast, altering C-terminal regions of the SNARE motifs (VAMP2 A81G/A82G or Δ85–94; syntaxin V237G/E238G/H239G, T251G/K252G, or Δ255–264) did not affect the stability of the template complex (Figure 4 and Table 1). Thus, syntaxin regions C-terminal to the +3 layer, and VAMP2 regions C-terminal to the +6 layer, are likely disordered in the template complex.

### Template complex facilitates SNARE assembly

**Figure 5.**
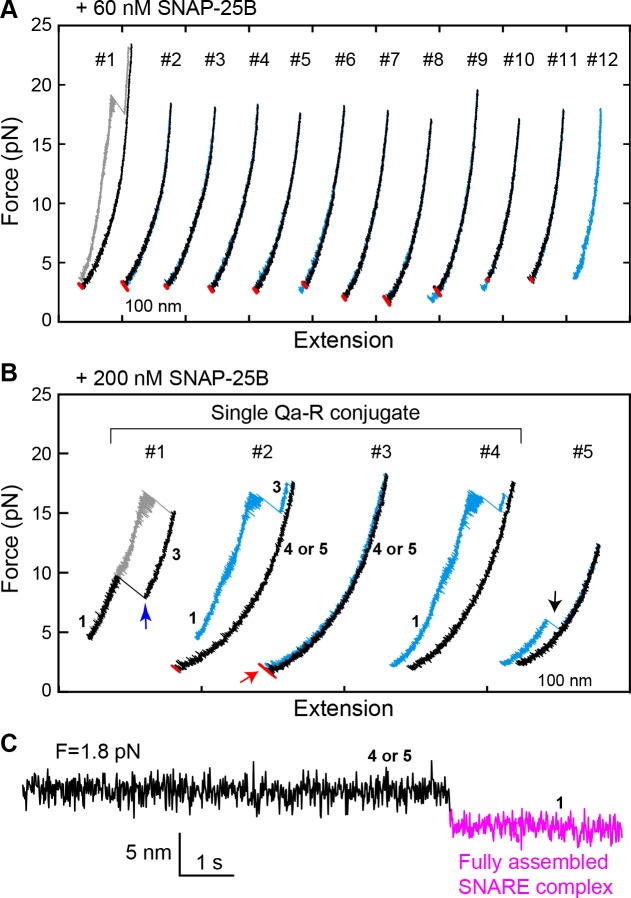
Spontaneous SNARE assembly in the absence of Munc18-1 is inefficient. (**A**) Representative FECs obtained by consecutively pulling and relaxing a single Qa-R SNARE conjugate in the presence of 60 nM SNAP-25B (Figure 5—source data 1). No SNARE assembly is observed. (**B**) Representative FECs obtained in the presence of 200 nM SNAP-25B showing spontaneous SNARE assembly (#1–4, Figure 5—source data 2) and misassembly (#5). FECs #1–4 are from a single Qa-R SNARE conjugate. In FEC #1, the SNARE complex was relaxed just after the t- and v-SNAREs were unzipped to observe t-v zippering at low force (blue arrow). The black arrow marks disassembly of the misfolded SNARE complex. Throughout figures, red arrows indicate SNARE reassembly. (**C**) Extension-time trajectory exhibiting cooperative de novo SNARE assembly at a constant mean force (Figure 5—source data 3). Shown here is the spontaneous SNARE assembly observed in panel B marked by the red arrow. Throughout figures, the fully assembled SNARE state (state 1 or 8) is shown in magenta. 10.7554/eLife.41771.025Figure 5—source data 1.MATLAB figure corresponding to Figure 5A. 10.7554/eLife.41771.026Figure 5—source data 2.MATLAB figure corresponding to Figure 5B (FEC#1–4). 10.7554/eLife.41771.027Figure 5—source data 3.MATLAB figure corresponding to Figure 5C.

**Figure 6.**
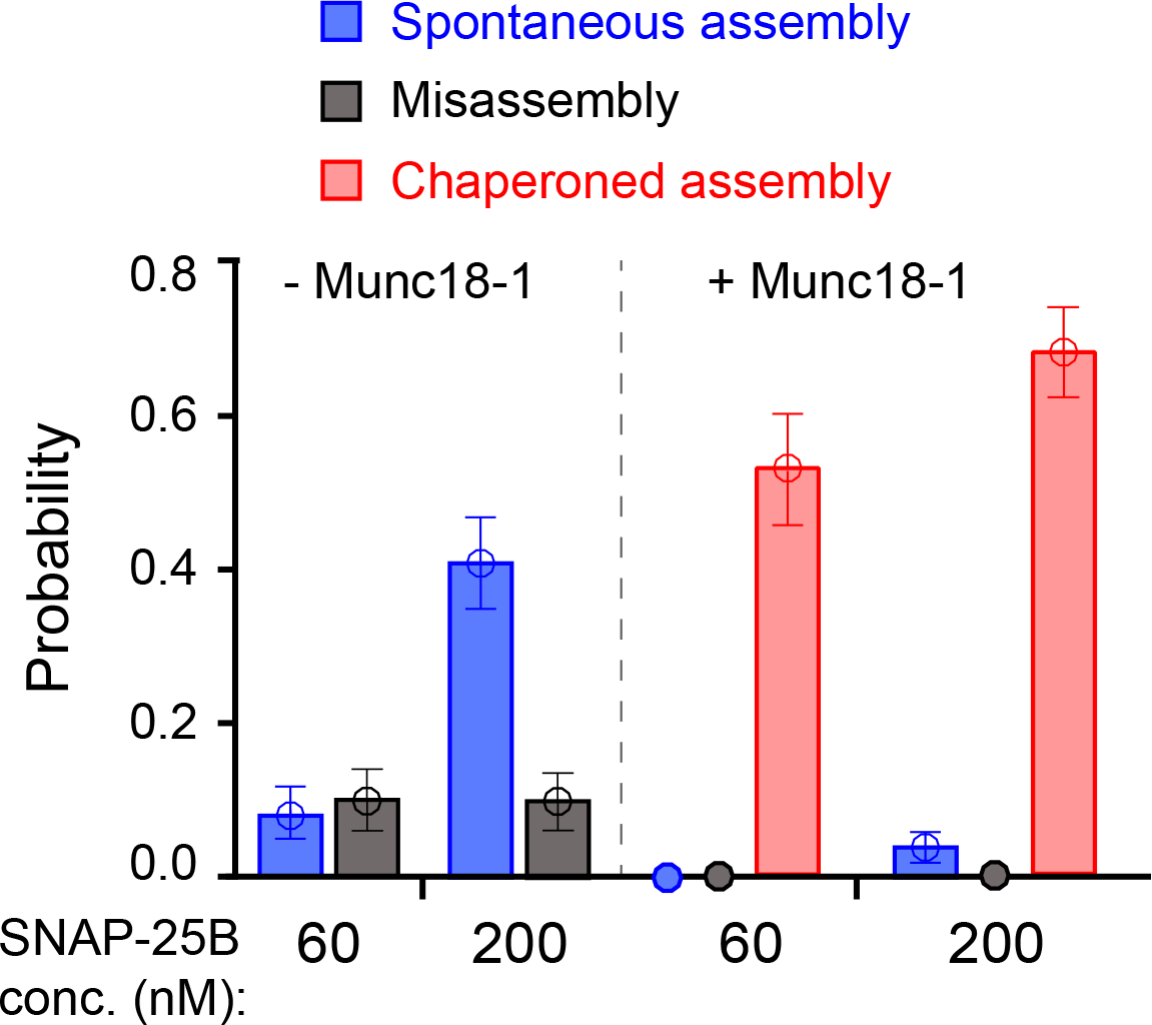
Comparison of SNARE assembly in the absence and presence of Munc18-1. Bars indicate probabilities of Munc18-1-independent or spontaneous SNARE assembly (blue), Munc18-1-chaperoned SNARE assembly (red), and SNARE misassembly (black). See also Figure 6—source data 1. 10.7554/eLife.41771.029Figure 6—source data 1.Data summary table for the results shown in Figure 6.Dataset 1. This dataset contains 18 MATLAB figures corresponding to Figures 2–10, as are listed below. Each plot or curve in a MATLAB figure has its associated data embedded. The data values for all data points in a plot can be obtained using MATLAB command get(plot_handle,’xdata’) or get(plot_handle,’ydata’), where plot_handle is a unique identifier of the plot.

To investigate a potential role for the template complex in SNARE assembly, we repeatedly relaxed and pulled the neuronal Qa-R-SNARE conjugate in the presence of SNAP-25B and, where indicated, Munc18-1. During relaxation, we held the Qa-R-SNARE conjugate at constant mean forces, typically around the equilibrium force of the template complex (Table 1), for up to 60 s to afford an opportunity for SNAP-25B binding and SNARE assembly. The probability of SNARE assembly per relaxation was measured to assess the efficiency of SNARE assembly. Properly assembled SNARE complexes were taken to be those that: (i) displayed the same extension at low force as the initial purified complex, and (ii) exhibited stepwise unfolding in subsequent rounds of pulling. In the presence of 60 nM SNAP-25B but no Munc18-1, the SNAREs rarely assembled, with a probability of only 0.08 per relaxation (Figures 5A and 6). Increasing the SNAP-25B concentration to 200 nM increased the assembly probability to 0.41 (Figure 5B, #2–4; Figure 6). This ‘spontaneous’ (i.e. Munc18-1-independent) SNARE assembly is observed as a single extension drop in the force range of 2–4 pN (Figure 5C). The t-SNARE complex forms in the same force range (Zhang et al., 2016b), indicating that the spontaneous SNARE assembly we observe here is likely limited by t-SNARE formation. Consistent with the interpretation that the t-SNARE complex is an obligate intermediate in spontaneous SNARE assembly, zippering between a pre-formed t-SNARE complex and the v-SNARE (t-v zippering) occurs at much higher force (Figure 5B, #1). Notably, SNAREs misassembled in the absence of Munc18-1 with a probability of ~0.1, as judged by premature unfolding of the incorrectly assembled complex at low force upon subsequent pulling (Figure 5B, #5; Figure 6).

**Figure 7.**
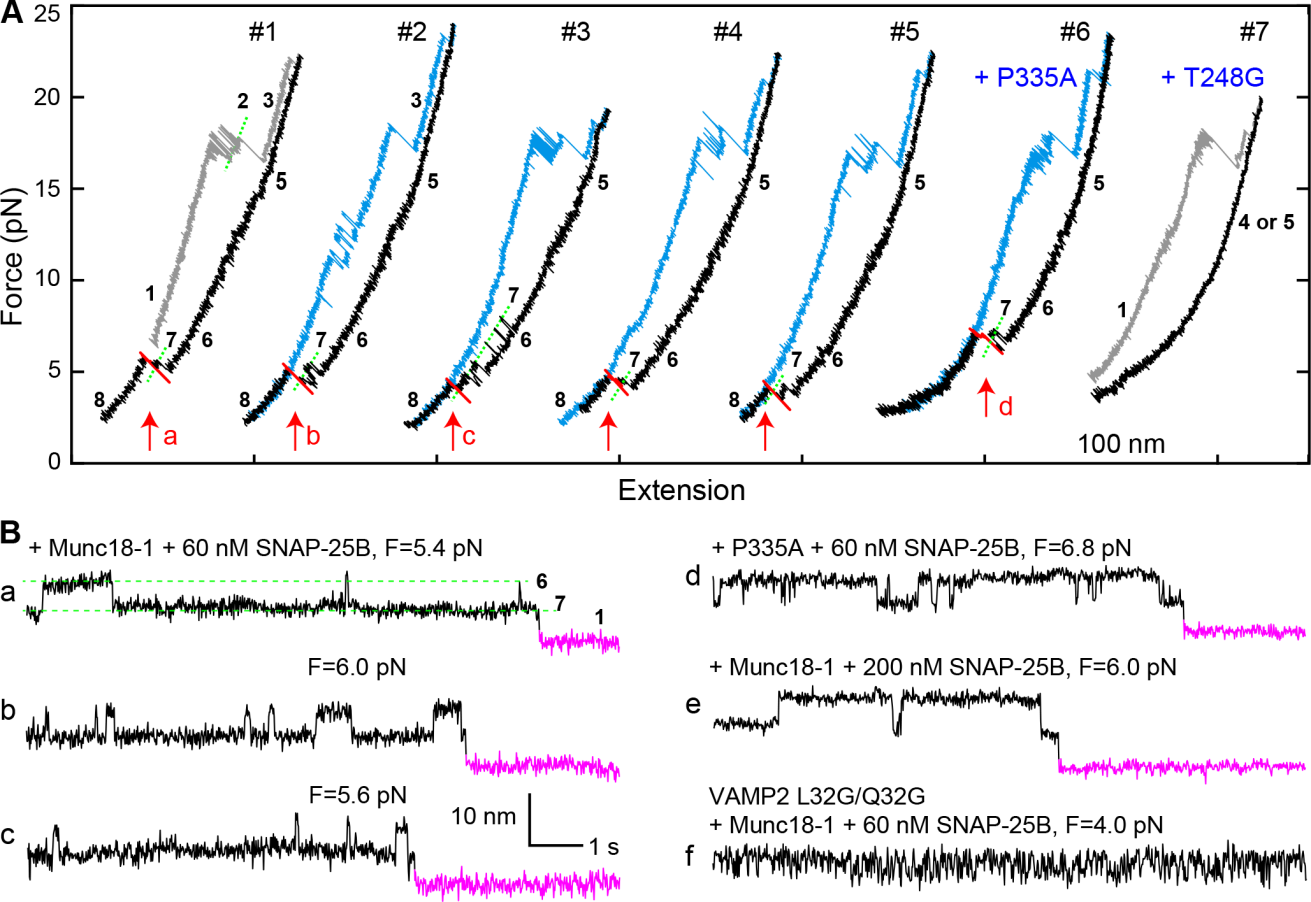
Template complex facilitates SNARE assembly. (**A**) Representative FECs obtained in the presence of 60 nM SNAP-25B and 2 µM WT Munc18-1 (#1–5 ) or Munc18-1 mutants P335A (#6) or T248G (#7). FECs #1–5 represent consecutive rounds of manipulation of a single Qa-R SNARE conjugate. See also Figure 7—figure supplement 1 and Figure 7—source data 1. (**B**) Extension-time trajectories at the indicated constant mean forces showing SNARE assembly. Traces a-d were extracted from FEC regions marked with correspondingly labeled red arrows in panel A. Trace f shows rapid template complex transitions without SNAP-25B binding. See also Figure 7—figure supplement 2 and Figure 7—source data 2. 10.7554/eLife.41771.033Figure 7—source data 1.MATLAB figure corresponding to Figure 7A (FECs #1–5). 10.7554/eLife.41771.034Figure 7—source data 2.MATLAB figure corresponding to Figure 7B.

The addition of 2 μM Munc18-1 significantly promoted SNARE assembly in the presence of 60 nM SNAP-25B. In a representative experiment, a single Qa-R SNARE conjugate assembled into a proper SNARE complex during each of five consecutive rounds of pulling and relaxation (Figure 7A, #1-#5). Overall, based on 67 pulling and relaxation FECs conducted using 15 Qa-R SNARE conjugates, proper assembly was observed with a probability of 0.53 per relaxation (Figure 6). However, SNARE assembly tended to occur consecutively: the conditional probability of observing one SNARE assembly event after another such event was 0.79 (N = 52) (Figure 7A, #1-#5), likely mediated by a single Munc18-1 molecule. Every SNARE assembly event was accompanied by a SNAP-25B-dependent, 5.5 ± 0.3 nm (N = 50) extension drop from an intermediate state (Figure 7B,a–c; Video 2). This intermediate had the same average extension relative to the unfolded state 5, the same equilibrium force, and the same response to mutations as the template complex (Figure 7A, #6–7; Figure 7B,d; Figure 7—figure supplements 1 and 2). We conclude that in the presence of Munc18-1, the pre-assembled template complex is required for SNAP-25B binding and SNARE assembly. This conclusion remains valid when the SNAP-25B concentration was increased to 200 nM (Figure 7B,e). In this case, the probability of Munc18-1-chaperoned SNARE assembly increased to 0.68 per relaxation (N = 38) (Figure 6). In addition, at this higher SNAP-25B concentration, we observed two instances of spontaneous SNARE assembly – that is, assembly not preceded by the formation of the template complex. Importantly, however, the probability of spontaneous assembly events in the presence of Munc18-1 (0.036) was more than ten-fold lower than the probability of spontaneous assembly events in the absence of Munc18-1 (0.41; see above). Thus, Munc18-1 both promotes SNARE assembly via the template complex and inhibits SNARE assembly via the t-SNARE complex (Figure 6).

**Figure 7—figure supplement 1.**
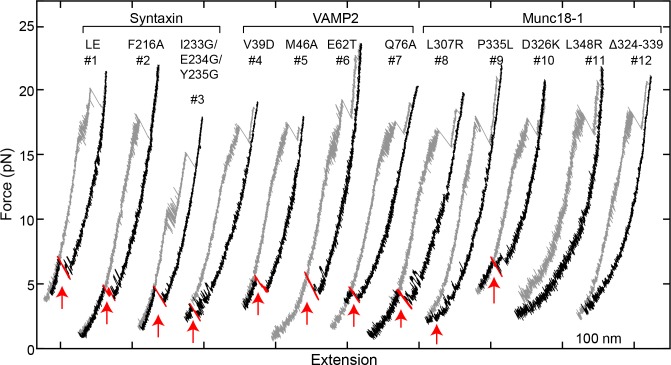
FECs obtained in the presence of 2 µM Munc18-1 and 60 nM SNAP-25B in the solution. Red arrows mark events of SNAP-25B binding and SNARE assembly. Note that syntaxin +2 layer mutation I233G/E234G/Y235G significantly weakens CTD zippering.

**Figure 7—figure supplement 2.**
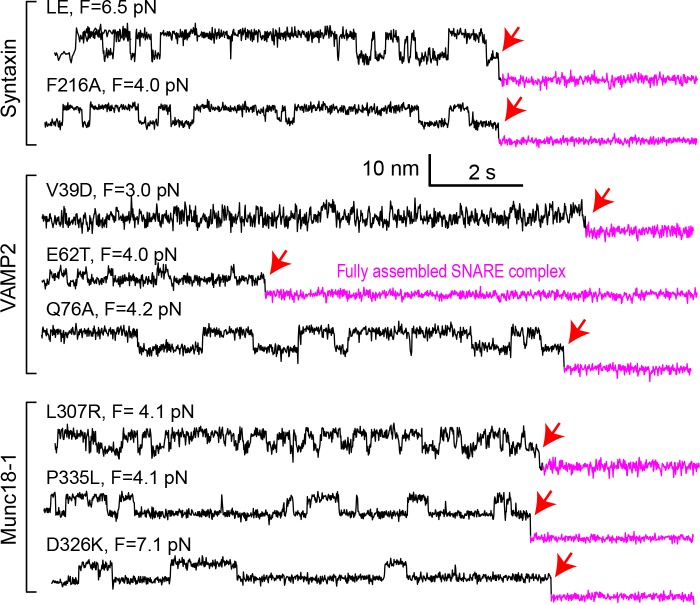
Extension-time trajectories at constant mean forces. The trajectories exhibit reversible folding and unfolding transitions of the mutant template complexes and irreversible SNAP-25 binding (indicated by red arrows).

**Figure 8.**
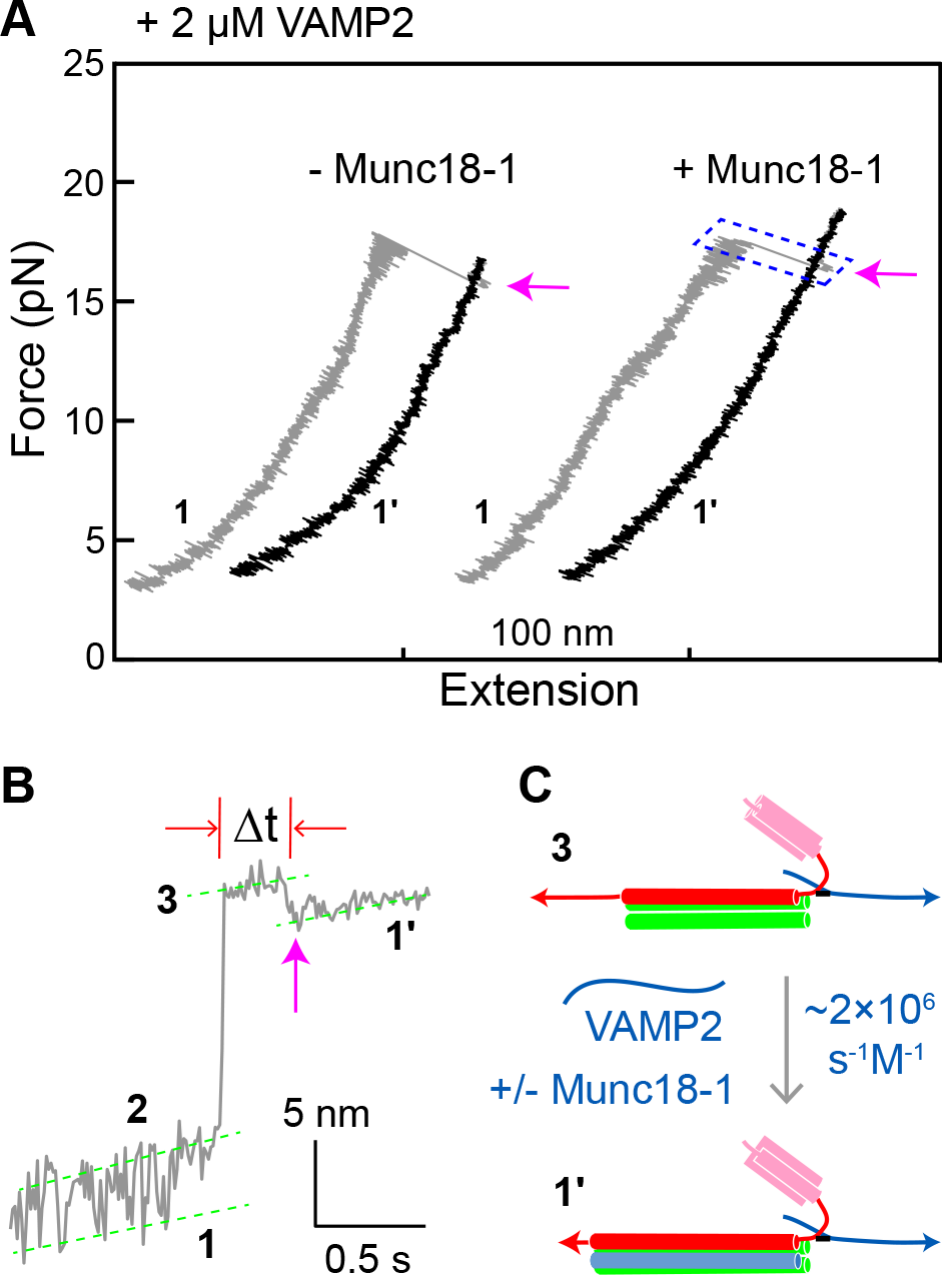
Munc18-1 does not significantly accelerate zippering between t- and v-SNAREs. (**A**) FECs obtained by pulling single WT SNARE complexes in 2 µM soluble VAMP2 in the absence or presence of 2 µM Munc18-1. Magenta arrows mark binding of the VAMP2 molecules in the solution to the t-SNARE complexes generated by unzipping the ternary SNARE complex (see panel C). See also Figure 8—source data 1. (**B**) Close-up view of an extension-time trajectory displaying VAMP2 binding in trans. The trajectory corresponds to the boxed pulling region in A. As observed previously (Ma et al., 2015; Zhang et al., 2016b), VAMP2 binding (indicated by the magenta arrow) induced folding of the disordered C-terminus of the t-SNARE complex, decreasing its extension by 2.3 ± 0.1 nm and generating state 1′ (see panel C). It took an average time (Δt) of ~0.3 s for the free VAMP2 in the solution to bind the t-SNARE complex. Note that the SNARE complexes in state 1′ and state 1 (Figure 2D) are pulled from different sites. See also Figure 8—source data 2. (**C**) Diagram illustrating VAMP2 induced t-SNARE folding and extension shortening. 10.7554/eLife.41771.036Figure 8—source data 1.MATLAB figure corresponding to Figure 8A. 10.7554/eLife.41771.037Figure 8—source data 2.MATLAB figure corresponding to Figure 8B.

**Video 2.** Template complex facilitates SNAP-25B binding and SNARE assembly. The extension at a constant mean force of 6.0 pN corresponding to trace b in Figure 7B and its associated state transition are simulated. For simplicity, only the right bead was simulated to move in response to SNARE conformational changes. In reality, the left bead moved synchronously with the right bead, but in an opposite direction, as shown in Video 1.

The template complex greatly accelerated proper SNARE assembly. SNAP-25B bound to the template complex with probabilities of 0.71 and 0.84 per relaxation at 60 nM and 200 nM SNAP-25B, respectively, yielding a binding rate constant of ~5 × 10^5^ M^−1^s^−1^. The rate constant is 25-fold greater than that observed in the absence of Munc18-1 (~2 × 10^4^ M^−1^s^−1^), presumably because Munc18-1 pre-aligns the N-terminal portions of the syntaxin and VAMP2 SNARE motifs for recognition by SNAP-25B. Consistent with this view, the VAMP2 −7 layer mutations L32G/L33G nearly abolished SNAP-25B binding (Figure 7B,f; Table 1). Notably, we did not observe any misassembly events in the presence of Munc18-1 (Figure 6). Thus, Munc18-1 enhanced the speed, and probably the accuracy, of SNARE assembly.

In most cases, the disassembly FECs of the Munc18-1-reassembled SNARE complexes were indistinguishable from those of the initial SNARE complexes (Figure 7A, compare blue curves in #3–5 to gray curve in #1), consistent with proper SNARE reassembly. 14% of the time, however, the subsequent pulling FEC revealed unfolding of the VAMP2 CTD at unusually low force of 4–14 pN (Figure 7A, #2). These are not misassembly events, because the SNARE complexes show stepwise NTD unfolding and t-SNARE unfolding identical to the properly assembled SNARE complexes. Further work is required to determine the conformation of these complexes.

### N-terminal regulatory domain of syntaxin stabilizes template complex

Once initiated, the reversible template complex transition (6↔7 or 5↔7) typically persisted for over 10 min at constant mean force, even after the free Munc18-1 in the solution was removed. We suspected that the persistent association between Munc18-1 and the Qa-R-SNARE conjugate was attributable to the NRD of syntaxin, as suggested by previous results (Burkhardt et al., 2008; Shen et al., 2010; Shen et al., 2007; Zhou et al., 2013). Indeed, NRD truncation (ΔNRD) reduced the probability of observing template complex formation from 0.5 to 0.08 (Figure 2C, #5), consistent with the idea that the NRD recruits Munc18-1. The average lifetime of the template complex formed by ΔNRD was also shorter (Figure 3I and Figure 3—figure supplement 3), indicating that the NRD stabilizes the template complex. Unexpectedly, addition of 2 μM NRD in trans was able to rescue the defect caused by ΔNRD: the template complex now formed efficiently (probability = 0.6, N = 35) at an equilibrium force close to that of the WT template complex, albeit with slower transition kinetics (Figure 3I and Figure 3—figure supplement 1). Thus, the NRD can bind to and stabilize the template complex in trans.

Next, we dissected the roles of different NRD regions. Removing the ‘N-peptide’ at the extreme N-terminus of the NRD (Figure 2B) destabilized the template complex (Figure 3I and 4; Table 1), while removing the three-helix bundle H_abc_ domain (ΔH_abc_) abolished template complex formation altogether (Table 1 and Figure 3—figure supplement 1). By contrast the ‘LE’ mutation (L165A/E166A in the linker region between the H_abc_ domain and the SNARE motif; see Figure 2B) (Dulubova et al., 1999), which promoted SNARE assembly as expected (Burkhardt et al., 2008; Gerber et al., 2008; Ma et al., 2011; Richmond et al., 2001), stabilized the template complex (Figures 3I and 4A). Taken together, our results imply that the NRD has a three-fold role in template complex formation. When syntaxin is closed (state 6' in Figure 2B), the NRD inhibits template complex formation (Figure 2—figure supplement 5), as also shown previously (Burkhardt et al., 2008). When syntaxin is partially open (state 6 in Figure 2D), the NRD recruits Munc18-1 for fast folding of the template complex. Finally, once the template complex has formed (state 7 in Figure 2D), the NRD plays a direct stabilizing role. The structural basis for this stabilizing role awaits further investigation.

### Munc18-1 inhibits t-SNARE complex formation

As noted above, Munc18-1 not only promotes SNARE assembly via the template complex, but also inhibits spontaneous SNARE assembly (i.e. assembly not preceded by template complex formation). Using a very similar experimental approach, we previously showed that spontaneous SNARE assembly proceeds by a different route (Gao et al., 2012; Zhang et al., 2016b): first, syntaxin binds SNAP-25B to form a t-SNARE complex; then, VAMP2 zippers with the t-SNARE complex in a process called t-v zippering (Gao et al., 2012; Zhang et al., 2016b). In the presence of Munc18-1, t-SNARE complexes were never observed. Thus, Munc18-1 appears to inhibit spontaneous SNARE assembly by suppressing formation of the t-SNARE complex intermediate.

Our results argue that Munc18-1 accelerates SNARE assembly by means of an on-pathway template complex intermediate. By contrast, a previous model instead proposed that Munc18-1 accelerates t-v zippering (Dawidowski and Cafiso, 2016; Jakhanwal et al., 2017; Shen et al., 2007; Zhang et al., 2016b). To address this possibility directly, we pulled ternary SNARE complexes to generate the t-SNARE complex (state 3) in the presence of 2 μM soluble VAMP2 (Figure 8A). The free VAMP2 molecule rapidly bound the t-SNARE complex (Figure 8B,C), with a binding rate constant (1.6 × 10^6^ M^−1^s^−1^) close to a previously published value (0.5 × 10^6^ M^−1^s^−1^) (Pobbati et al., 2006). The binding constant was little changed (2.0 × 10^6^ M^−1^s^−1^) by the addition of 2 µM Munc18-1. The average t-v zippering force was ~10 pN with or without Munc18-1 (Figure 5B, #1, blue arrow). Thus, we find no evidence to support the model that Munc18-1 promotes t-v zippering.

### Function-altering and phosphomimetic mutations

**Figure 9.**
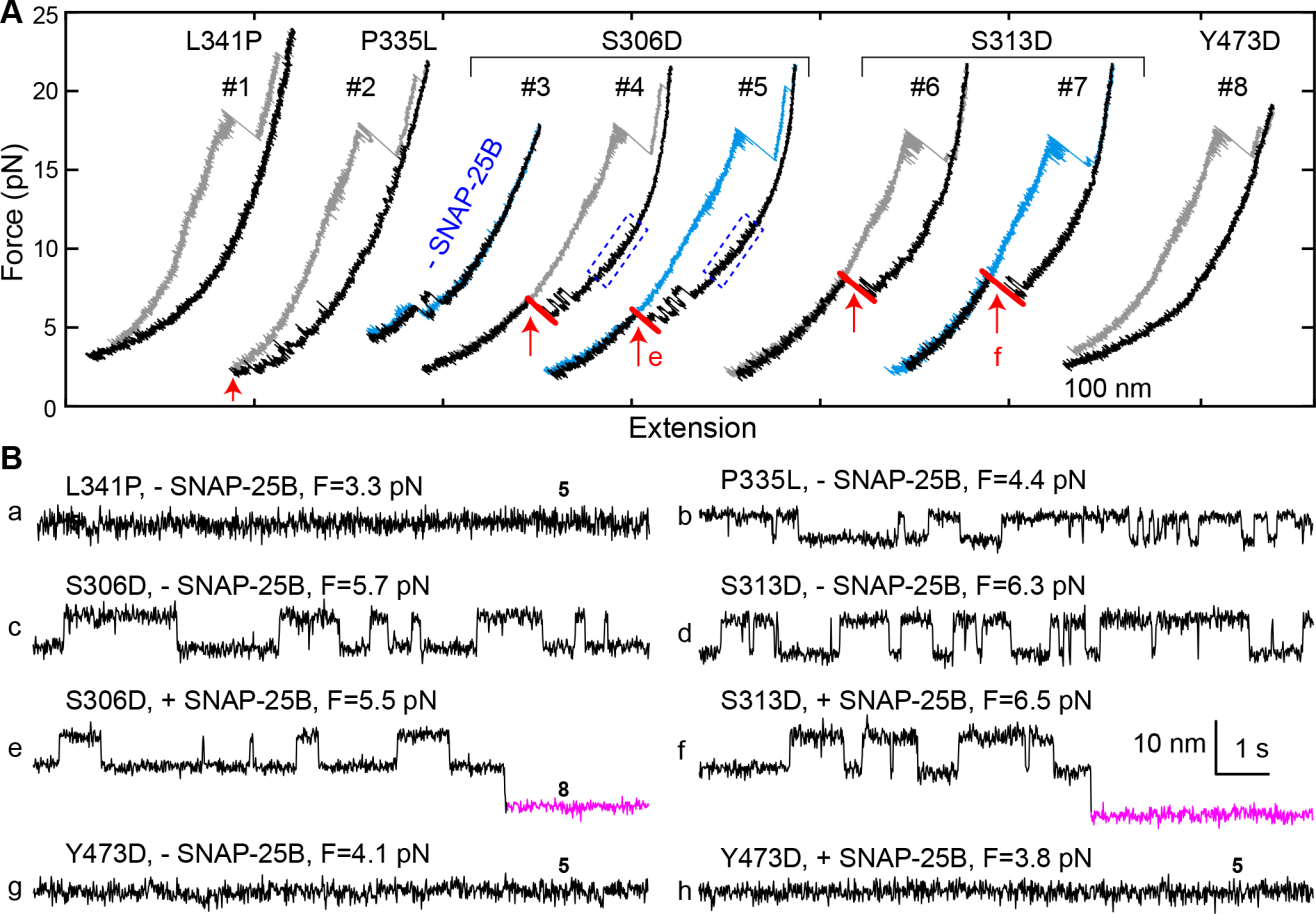
Munc18-1 phosphomimetic and disease mutations altered chaperoned SNARE assembly. (**A**) FECs for Munc18-1 mutations with 0 nM (#3) or 60 nM (others) SNAP-25B. See also Figure 9—source data 1. (**B**) Extension-time trajectories at the indicated constant mean forces, some of which (**e and f**) are extracted from panel A. In panels a, g, and h, no template complex formation is observed. See also Figure 9—source data 2. 10.7554/eLife.41771.040Figure 9—source data 1.MATLAB figure corresponding to Figure 9A (FECs #3–7). 10.7554/eLife.41771.041Figure 9—source data 2.MATLAB figure corresponding to Figure 9B.

In examining a large number of SNARE and SM mutations (Figure 4A and Table 1), we found that modifications known to compromise SNARE assembly, membrane fusion, and/or neurotransmitter release (Munch et al., 2016; Parisotto et al., 2014; Shen et al., 2007; Walter et al., 2010; Zhou et al., 2013) invariably destabilized the template complex (Figure 4A, red). Munc18-1 I341P and P335L are two examples of hundreds of Munc18-1 and SNARE mutations associated with epilepsy and other disorders (Stamberger et al., 2016). P335L destabilized, and I341P abrogated altogether, the template complex, leading to impaired Munc18-1-chaperoned SNARE assembly (Figure 9A, #1–2; Figure 9B,a,b; Figure 4A, purple). Conversely, mutations known to increase SNARE assembly, membrane fusion, and/or neurotransmitter release (Genc et al., 2014; Gerber et al., 2008; Munch et al., 2016; Parisotto et al., 2014; Sitarska et al., 2017) displayed enhanced template complex stability (Figure 4A, green bars). This correlation establishes that the template complex is an important intermediate for membrane fusion in vivo.

The phosphorylation of Munc18 proteins regulates neurotransmitter release and insulin secretion (de Jong and Verhage, 2009; Genc et al., 2014; Jewell et al., 2011; Meijer et al., 2018). To explore the mechanism(s) underlying these observations, we examined three phosphomimetic mutations, indicated by arrows in Figure 4A. Phosphorylation of domain 3a residues Ser 306 and Ser 313 (Figure 2A–B) enhances neurotransmitter release and contributes to short-term memory (Genc et al., 2014). Correspondingly, the phosphomimetic mutations S306D and S313D each stabilized the template complex (Figure 4A; Figure 9A, #3–7) and increased the probabilities of template formation and SNARE assembly (Table 1; Figure 9B,c–f). Conversely, the phosphomimetic mutation Y473D, which abrogates membrane fusion in vivo (Meijer et al., 2018), destabilized the template complex (Figure 4A; Figure 9A, #8; Figure 9B,g–h) and reduced the probabilities of template formation and SNARE assembly (Table 1). Tyr 473 is located immediately adjacent to the predicted binding pocket for the +6 layer Phe of VAMP2 (Figure 3F) and likely plays a significant role in VAMP2 binding. Taken together, these results suggest that Munc18-1 phosphorylation regulates synaptic vesicle fusion by modulating the stability of the template complex.

Many of the mutations that we tested cause modest changes in the unfolding energy of the template complex (Figure 4A) but, seemingly paradoxically, cause large changes in membrane fusion activity (Genc et al., 2014; Munch et al., 2016; Parisotto et al., 2014; Sitarska et al., 2017; Stamberger et al., 2016). To appreciate the impact of template complex stability on membrane fusion, we estimated the relative rate of membrane fusion mediated by the mutant or WT SNARE-Munc18-1 complex. Our estimation contains three assumptions. First, the template complex is a rate-limiting intermediate for SNARE assembly, and thus the overall rate of SNARE assembly is proportional to the equilibrium probability of the template complex determined by the Boltzmann distribution. Second, the mutation only affects the stability of the template complex, but not SNARE zippering, nor binding of any other regulatory proteins to the SNARE-Munc18-1 complex. Finally, we assume that the overall rate of membrane fusion or exocytosis is proportional to the overall rate of SNARE assembly. With these assumptions, the overall rate of membrane fusion k is proportional to exp(G)×r, where G and r are the unfolding energy of the template complex and the rate of SNAP-25B binding to the template complex, respectively. Consequently, the overall rate of membrane fusion mediated by a mutant SNARE-Munc18-1 complex $k_{m}$ relative to that of the WT complex $k_{WT}$ can be calculated as

(1) $$\frac{k_{m}}{k_{WT}}=\frac{r_{m}}{r_{WT}}\exp\left(G_{m}-G_{WT}\right),$$

where the subscripts m and WT represent mutant and wild-type, respectively. Using this equation, we computed the relative rate of membrane fusion for all of the mutants we tested (Figure 4B). Our measurements predict that Munc18-1 mutation P335A increases the overall rate of membrane fusion by 2.9 (±0.9) fold, in good agreement with the observed enhancement of ~2 fold for evoked release in the cell and of 4–5 fold in liposome-liposome fusion in vitro (Munch et al., 2016; Parisotto et al., 2014). For Munc18-1 D326K, we predict an increase in the fusion rate by 5 (±1) fold, consistent with the activation of membrane fusion observed in vitro in the absence of calcium triggering: ~4 fold for lipid mixing and ~7 fold for content mixing (Sitarska et al., 2017). By contrast, both reconstituted membrane fusion and our prediction show that Munc18-1 L348R abolished membrane fusion (Parisotto et al., 2014; Sitarska et al., 2017). In conclusion, the concordance between the effect of mutations on membrane fusion or neurotransmitter release and on the stability of the template complex strongly suggests that the template complex is a physiologically relevant intermediate in SNARE assembly.

Finally, we note that VAMP2 and syntaxin mutations, in addition to changing the stability of the template complex, can influence subsequent SNARE zippering. For example, VAMP2 F77A destabilizes both the template complex and the C-terminal region of the SNARE complex (Figure 3—figure supplement 1, FEC #25). Layer mutations C-terminal to the +3 layer in syntaxin and the +6 layer in VAMP2 also destabilize the C-terminal region of the SNARE complex, but without affecting the template complex (Figure 4A). In general, template complex stability does not predict the impact of given mutation on SNARE zippering. Nonetheless, our measurements make it possible to dissect the respective contributions of Munc18-1 chaperoning and SNARE zippering to the dynamics of membrane fusion.

### Template mechanism is conserved among SM proteins

**Figure 10.**
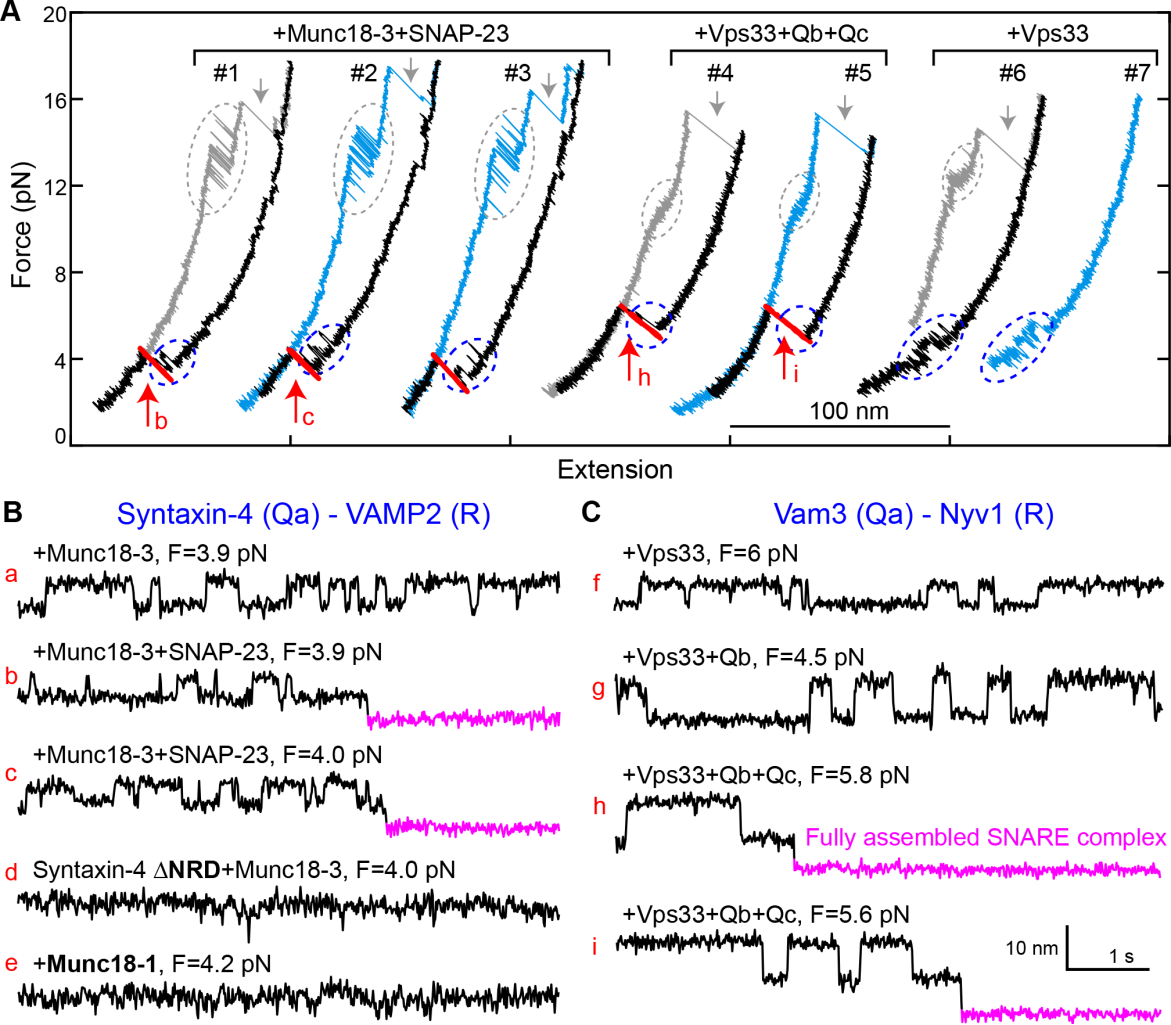
Munc18-3 and Vps33 catalyze SNARE assembly via template complexes. (**A**) FECs of the Munc18-3 or Vps33 cognate Qa-R SNARE conjugate in the presence of the indicated protein(s). See also Figure 10—source datas 1 and 2. (**B–C**) Extension-time trajectories at the indicated constant mean forces, some of which (b, c, h, and i) are extracted from panel A. See also Figure 10—source datas 3 and 4. 10.7554/eLife.41771.046Figure 10—source data 1.MATLAB figure corresponding to Figure 10A (FECs #1–3). 10.7554/eLife.41771.047Figure 10—source data 2.MATLAB figure corresponding to Figure 10A (FECs #4–7). 10.7554/eLife.41771.048Figure 10—source data 3.MATLAB figure corresponding to Figure 10B (traces a-e). 10.7554/eLife.41771.049Figure 10—source data 4.MATLAB figure corresponding to Figure 10C (traces g-i).

To generalize our findings, we investigated two other SM proteins, Munc18-3 and Vps33 (Figure 2—figure supplement 1). Munc18-3 and its cognate SNAREs syntaxin 4 (Qa), VAMP2 (R), and SNAP-23 (Qbc) mediate fusion of glucose transporter 4- (GLUT4-) containing vesicles with the plasma membrane, promoting glucose uptake (Bryant and Gould, 2011; Yu et al., 2013). Vps33 and its cognate vacuolar SNAREs Vam3 (Qa), Nyv1 (R), Vti1 (Qb), and Vam7 (Qc) mediate membrane fusion in endo-lysosomal trafficking (Wickner, 2010). Like other SNARE complexes (Zorman et al., 2014), the GLUT4 and vacuolar SNARE complexes disassembled stepwise via one or more partially-zippered intermediates (Figure 10A). In the absence of SM proteins, spontaneous SNARE assembly was inefficient, with a probability per relaxation of 0.02 for GLUT4 SNAREs (60 nM SNAP-23) and of 0.04 for vacuolar SNAREs (1 µM Vti1 and 1 μM Vam7) (Figure 10—figure supplement 1). In the presence of 2 µM Munc18-3 or 0.4 µM Vps33, the probability of SNARE assembly increased to 0.44 for Munc18-3 and to 0.65 for Vps33 (Figure 10A, #1–5). Thus, both SM proteins strongly enhance the rate of SNARE assembly. All of the more than 50 Munc18-3-mediated SNARE assembly events we observed in our experiments were mediated by the corresponding template complex (blue ovals in Figure 10A, #1–3; Figure 10B,a–c). The Munc18-3 template complex displayed a stability (4.3 ± 0.2 k_B_T) and an extension relative to the unfolded state similar to those of the Munc18-1 template complex. In addition, the Munc18-3 template complex depended on the NRD of syntaxin 4, as NRD truncation reduced the probability of observing the template complex transition to 0.03 (Figure 10B,d). Thus, Munc18-3 and Munc18-1 are quantitatively similar in their ability to chaperone cognate SNARE complex assembly. Importantly, neither Munc18-1 nor Munc18-3 could catalyze the assembly of the other’s cognate SNAREs (Figure 10B,e).

**Figure 10—figure supplement 1.**
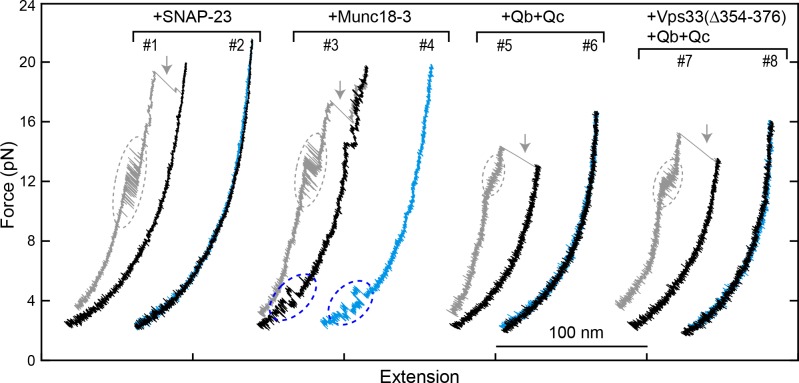
FECs obtained by pulling and relaxing a single syntaxin-4-VAMP2 conjugate (#1–4) or Vam3-Nyv1 conjugate (#5–8) in the presence of the indicated protein or proteins. SNARE CTD transitions and template complex transitions are marked by gray and blue ovals, respectively. Gray arrows indicate SNARE unzipping. Vps33(Δ354–376) is analogous to Munc18-1 Δ324–339 and is inactive in vivo and in vitro (Baker et al., 2015).

**Figure 10—figure supplement 2.**
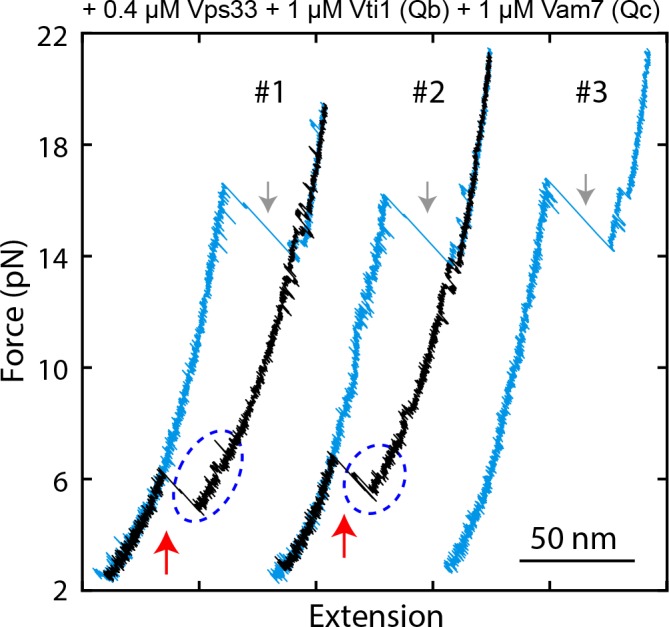
FECs displaying Vps33-catalyzed vacuolar SNARE assembly, marked by red arrows. Gray arrows indicate SNARE unzipping. Template complex transitions are marked by blue ovals.

**Figure 10—figure supplement 3.**
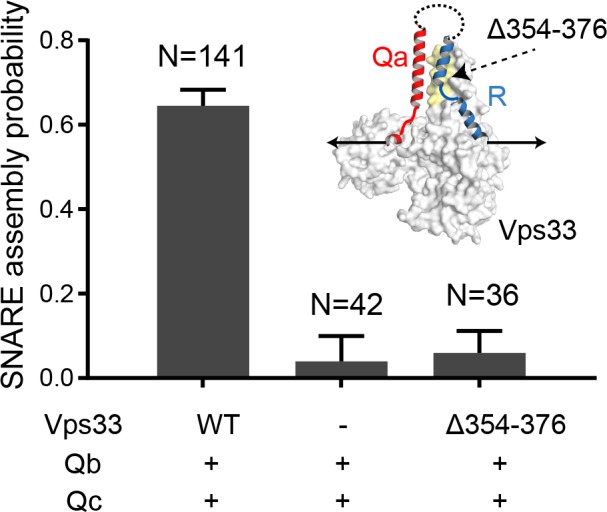
Probabilities of vacuolar SNARE assembly per relaxation under different conditions. The insert shows the pulling direction and the region of the Vps33 truncation (yellow).

We also observed a Vps33-mediated template complex in the absence of Qb- and Qc-SNAREs (blue ovals in Figure 10A, #6–7; Figure 10C,f). Relaxing the Qa-R-SNARE conjugate in the presence of both Vps33 and the Qb-SNARE increased the extension change associated with the template complex transition from 4–6 nm to 7–9 nm (Figure 10C, compare trace g to trace f), indicating that the Qb-SNARE induced further folding of the templated SNAREs. Relaxing the Qa-R-SNARE conjugate in the presence of both the Qb- and Qc-SNAREs triggered assembly of the full SNARE complex from the template complex (Figure 10A, #4–5; Figure 10—figure supplements 2 and 3). Taken together, these results indicate that Munc18-3 and Vps33 catalyze SNARE assembly by templating SNARE folding and association in a manner analogous to that observed for Munc18-1, in strong support of a conserved templating mechanism underlying SM protein function.

## Discussion

Using geometrically faithful single-molecule experiments, we have mapped out a new pathway for the assembly of neuronal SNARE complexes. As suggested previously (Baker et al., 2015; Sitarska et al., 2017), the key intermediate is a template complex in which the SM protein Munc18-1 serves as the template to arrange the Qa-SNARE syntaxin and the R-SNARE VAMP2 in a Y-shaped conformation with aligned NTDs and splayed CTDs. Although the first 3–4 layers of the NTDs are not expected to interact directly with the template, they nonetheless appear to be properly zippered in the template complex. Our experiments further indicate that the Qbc-SNARE SNAP-25 binds rapidly to the template complex, presumably by recognizing the properly aligned NTDs of the Qa- and R-SNAREs. Finally, full zippering happens in a single, apparently cooperative transition. Like most enzymatic intermediate states, the template complex is relatively unstable (see Materials and methods for further analysis), preventing it from functioning as a kinetic trap. Nevertheless, our experiments show that it is an obligatory and productive intermediate, which promotes both the speed and the accuracy of SNARE assembly. In addition, the extensive SM-SNARE interactions within the template complex presumably help to prevent the formation of non-cognate SNARE complexes, thereby enhancing the specificity of SNARE pairing (Shen et al., 2007).

Our data appear to be inconsistent with an alternative model in which Munc18-1 binds to and activates the t-SNARE complex to promote t-v zippering (Dawidowski and Cafiso, 2016; Jakhanwal et al., 2017) (Figure 1, upper pathway). We found that Munc18-1 inhibited t-SNARE complex formation and minimally affected t-v zippering, consistent with previous reports (Ma et al., 2013; Pobbati et al., 2006; Zhang et al., 2015). Shen et al. found that fusion between t-liposomes (containing syntaxin:SNAP-25 complexes) and v-liposomes (containing VAMP2) was only stimulated by Munc18-1 after all three were preincubated at 4°C for 3 hr (Shen et al., 2007). This preincubation, under conditions that prevent fusion, was presumably needed to allow formation of the Munc18-1-stabilized partially-zippered SNARE complexes we previously observed (Ma et al., 2015) (Figure 1, state v). Preincubating Munc18-1 with t-liposomes alone resulted in little stimulation (Shen et al., 2007), inconsistent with the formation of an activated Munc18-1:t-SNARE complex. Moreover, t-SNARE complexes, because they are vulnerable to the ubiquitous SNARE disassembly machinery NSF/SNAP (Lai et al., 2017; Yavuz et al., 2018), do not appear to represent plausible intermediates in physiological SNARE assembly pathways. Finally, in vivo imaging of SNARE proteins during exocytosis suggested that SNAP-25 is recruited to the fusion site and undergoes a conformational change immediately prior to membrane fusion (Gandasi and Barg, 2014; Zhao et al., 2013). A stable and pre-formed t-SNARE complex is unlikely to undergo the large SNAP-25 conformational change observed in these experiments (Zhang et al., 2016b).

Our results identify a new role for the NRD of syntaxin in stabilizing the template complex. The stabilizing effect of the NRD is partitioned between its N-peptide and its H_abc_ domain (Figure 2A–B; Figure 4). The stabilizing effect of the N-peptide, which binds to a distal site on Munc18-1 (Burkhardt et al., 2008), is unsurprising, as the N-peptide has long been thought to promote interactions between Munc18-1 and partially or completely folded SNARE complexes (Dulubova et al., 2007; Ma et al., 2015; Shen et al., 2007). However, the stabilizing effect of the H_abc_ domain, even when it is added in trans, is unexpected. This role adds to the others that have been ascribed to the syntaxin NRD and that have complicated efforts to elucidate the physiological neuronal SNARE assembly pathway (Meijer et al., 2012; Shen et al., 2010; Zhou et al., 2013). By contrast, the Qa-SNARE Vam3 does not adopt a closed conformation (Dulubova et al., 2001), a simplifying feature that prompted us to omit its NRD from both our earlier crystallographic studies (Baker et al., 2015) and from the single-molecule experiments reported here. Fortunately, the Vps33 template complex was observable in the absence of the Qa-SNARE NRD (Figure 10). Thus, whereas template complexes appear to be a general feature of SM-mediated SNARE assembly, their stabilization via Qa-SNARE NRDs may represent a more specialized elaboration.

Other factors involved in neurotransmitter release may impinge upon the template complex we have identified. For example, Munc13-1 plays important roles in opening syntaxin and promoting proper SNARE complex assembly (Lai et al., 2017; Ma et al., 2011; Yang et al., 2015). The opener function of Munc13-1 was circumvented in our studies by two orthogonal strategies, each of which precludes full syntaxin closure. Notably, however, fully closed syntaxin was only marginally more stable than the template complex (7.2 ± 0.2 k_B_T vs 5.2 ± 0.2 k_B_T; Figure 2—figure supplement 4). Given that Munc13-1 binds weakly to both syntaxin and VAMP2 at sites likely complementary to those involved in Munc18-1 binding (Lai et al., 2017; Sitarska et al., 2017; Wang et al., 2017), it is attractive to hypothesize that Munc13-1 exerts both its syntaxin opening and SNARE proofreading activities by binding to and stabilizing the template complex (Figure 1, from state i to state iv). Additional factors including complexin and synaptotagmin likely capture the SNARE complex downstream of the template complex, for example by binding to the partially zippered SNARE complex (state v), thereby imposing further regulatory constraints – especially calcium triggering – on synaptic vesicle fusion (Brunger et al., 2018).

The finding that several SM proteins – Munc18-1, Munc18-3, and Vps33 – all catalyze SNARE assembly via a template complex confirms that this is a key conserved function of SM proteins. SNARE zippering, because it involves the coupled folding and assembly of four intrinsically disordered SNARE motifs, is inefficient (Brunger, 2005; Lai et al., 2017). SM proteins, by increasing both the rate and fidelity of SNARE assembly, are likely to be key factors for the control of membrane fusion in vivo. Templated assembly may also resist the disassembly activity of NSF/SNAP.

We propose a working model, using neuronal exocytosis as an example, that places our results in the context of the full fusion machinery (Figure 1). First, SNAREs and SM proteins are recruited to, and thereby concentrated at, the future site of membrane fusion during vesicle docking (Figure 1, state i). Munc13-1 helps bridge vesicle and plasma membranes and recruit SNAREs, and catalyzes opening of the closed syntaxin (state ii) (Lai et al., 2017; Ma et al., 2011; Ma et al., 2013). Subsequently, Munc18-1 binds to the R-SNARE to form the template complex (iv), which may be further stabilized by Munc13-1. Binding of SNAP-25 generates a partially-zippered SNARE complex stabilized by Munc18-1 (state v). Synaptotagmin and complexin likely associate with the partially zippered SNARE complex, stabilizing it in a primed trans-SNARE complex in preparation for calcium-triggered exocytosis (Südhof and Rothman, 2009). Finally, calcium triggers fast CTD zippering and Munc18-1 displacement (state vi), inducing membrane fusion.

## Materials and methods

**Key resources table:** 

Reagent type (species) or resource	Designation	Source or reference	Identifiers	Additional information
Strain, strain background (*species*)	BL21 Gold (DE3) competent cells	Agilent echnologies	Cat#230132	
Commercial assay or kit	BirA-500: BirA biotin-protein ligase standard reaction kit	Avidity	Cat#BirA500	
Chemical compound, drug	dNTP mix (10 mM)	Invitrogen	Cat#18427013	
Chemical compound, drug	2,2'-dithiodipyridine disulfide (DTDP)	Sigma-Aldrich	CAS#2127-03-9	
Chemical compound, drug	Protease inhibitor cocktail tablet, EDTA free	Roche	Cat#05892791001	
Peptide, recombinant protein	Catalase from bovine liver	Sigma-Aldrich	CAS#9001-05-2	
Peptide, recombinant protein	Glucose Oxidase from *Aspergillus* *niger*	Sigma-Aldrich	CAS#9001-37-0	
Software, algorithm	LabVIEW VIs	(Gao et al., 2012)		instrument control, data acquisition, and data analysis
Software, algorithm	MATLAB scripts	(Gao et al., 2012) (Rebane et al., 2016)		data analysis
Software, algorithm	Geneious	Geneious		DNA primer design
Software, algorithm	GraphPad Prism7	GraphPad Software		
Other	Micro Bio- spin six columns	Bio-RAD	Cat#732–6221	
Other	Ni Sepharose 6 Fast Flow	GE healthcare Lifesciences	Cat#17531801	
Other	Anti-digoxigenin antibody coated polystyrene particles	Spherotech	Cat#DIGP-20–2	2.1 µm, called DIG beads
Other	Streptavidin-coated polystyrene particles	Spherotech	Cat#SVP-15–5	1.8 µm
Other	Customized glass tubing: bead dispenser tubes with 100 µm outer diameter (OD) and 25 µm inner diameter (ID)	King Precision Glass, Inc		
Other	Polyethylene tubing PE50	Becton Dickinson	Cat# 22–270835	
Other	Dual optical trap setup	(Gao et al., 2012)		

### SNARE constructs

The cytoplasmic domains of rat neuronal SNAREs, and the SNARE motifs of *C. thermophilum* vacuolar SNAREs, were used. Their sequences are listed below and their domains and crosslinking sites are shown in Figure 2—figure supplement 1. In the sequences below, numbers in parenthesis after each construct name indicate the amino acid numbering in the original protein sequence if there is any truncation, followed by the mutated amino acids, if any, which are also colored red in the sequence. The amino acids in the zero layer are colored blue. Extra sequences, including linker sequences, are underlined, with Avi-tags or cysteine residues used for crosslinking shown in bold.

### Vamp2 (1–96, N29C)

MSATAATVPPAAPAGEGGPPAPPPNLTS**C**RRLQQTQAQVDEVVDIMRVNVDKVLERDQKLSELDDRADALQAGASQFETSAAKLKRKYWWKNLKMMGGSGNGSGGL**C**TPSRGGDYKDDDDK

### Syntaxin-1A (1–265, R198C, C145S)

MKDRTQELRTAKDSDDDDDVTVTVDRDRFMDEFFEQVEEIRGFIDKIAENVEEVKRKHSAILASPNPDEKTKEELEELMSDIKKTANKVRSKLKSIEQSIEQEEGLNRSSADLRIRKTQHSTLSRKFVEVMSEYNATQSDYRER**S**KGRIQRQLEITGRTTTSEELEDMLESGNPAIFASGIIMDSSISKQALSEIET**C**HSEIIKLENSIRELHDMFMDMAMLVESQGEMIDRIEYNVEHAVDYVERAVSDTKKAVKYQSKARRKKGGSGNGGSGS**GLNDIFEAQKIEWHE**

### Syntaxin-1A (1–265, I187C, C145S)

MKDRTQELRTAKDSDDDDDVTVTVDRDRFMDEFFEQVEEIRGFIDKIAENVEEVKRKHSAILASPNPDEKTKEELEELMSDIKKTANKVRSKLKSIEQSIEQEEGLNRSSADLRIRKTQHSTLSRKFVEVMSEYNATQSDYRER**S**KGRIQRQLEITGRTTTSEELEDMLESGNPAIFASGIIMDSS**C**SKQALSEIETRHSEIIKLENSIRELHDMFMDMAMLVESQGEMIDRIEYNVEHAVDYVERAVSDTKKAVKYQSKARRKKGGSGNGGSGS**GLNDIFEAQKIEWHE**

### Syntaxin-1A, ΔNRD (187–265, R198C)

ISKQALSEIET**C**HSEIIKLENSIRELHDMFMDMAMLVESQGEMIDRIEYNVEHAVDYVERAVSDTKKAVKYQSKARRKKGGSGNGGSGS**GLNDIFEAQKIEWHE**

### Syntaxin-1A, ΔH_abc_ (Δ27–146, R198C)

MKDRTQELRTAKDSDDDDDVTVTVDRTSGRIQRQLEITGRTTTSEELEDMLESGNPAIFASGIIMDSSISKQALSEIET**C**HSEIIKLENSIRELHDMFMDMAMLVESQGEMIDRIEYNVEHAVDYVERAVSDTKKAVKYQSKARRKKGGSGNGGSGS**GLNDIFEAQKIEWHE**

### SNAP-25B (C85S, C88S, C90S, C92S)

MAEDADMRNELEEMQRRADQLADESLESTRRMLQLVEESKDAGIRTLVMLDEQGEQLERIEEGMDQINKDMKEAEKNLTDLGKF*S*GL*S*V*S*P*S*NKLKSSDAYKKAWGNNQDGVVASQPARVVDEREQMAISGGFIRRVTNDARENEMDENLEQVSGIIGNLRHMALDMGNEIDTQNRQIDRIMEKADSNKTRIDEANQRATKMLGSG

### Syntaxin-4 (1–273, Q194C)

MRDRTHELRQGDNISDDEDEVRVALVVHSGAARLSSPDDEFFQKVQTIRQTMAKLESKVRELEKQQVTILATPLPEESMKQGLQNLREEIKQLGREVRAQLKAIEPQKEEADENYNSVNTRMKKTQHGVLSQQFVELINKSNSMQSEYREKNVERIRRQLKITNAGMVSDEELEQMLDSGQSEVFVSNILKDT**C**VTRQALNEISARHSEIQQLERSIRELHEIFTFLATEVEMQGEMINRIEKNILSSADYVERGQEHVKIALENQKKARKKKGGSGNGGSGS**GLNDIFEAQKIEWHE**

### Syntaxin-4, ΔNRD (191–273, R206C)

GKDTQVTRQALNEISA**C**HSEIQQLERSIRELHEIFTFLATEVEMQGEMINRIEKNILSSADYVERGQEHVKIALENQKKARKKKGGSGNGGSGS**GLNDIFEAQKIEWHE**

### SNAP-23 (C79S, C80S, C83S, C85S, C87S)

MDDLSPEEIQLRAHQVTDESLESTRRILGLAIESQDAGIKTITMLDEQGEQLNRIEEGMDQINKDMREAEKTLTELNKSSGLSVSPSNRTKNFESGKNYKATWGDGGDSSPSNVVSKQPSRITNGQPQQTTGAASGGYIKRITNDAREDEMEENLTQVGSILGNLKNMALDMGNEIDAQNQQIQKITEKADTNKNRIDIANTRAKKLIDS

### Nyv1 (148-218)

GSS**C**GGGVENNGGDSINSVQREIEDVRGIMSRNIEGLLERGERIDLLVDKTDRLGGSAREFRLRSRGLKRKMWWKNVKGGSGNGSGGG**C**KAAA

### Vam3 (181-252)

GSS**C**GGGLILEREEEIRNIEQGVSDLNVLFQQVAQLVAEQGEVLDTIERNVEAVGDDTRGADRELRAAARYQKRARSRMGGSGNGSGLKNSGGSGSGGNRGGSDSGGSG**GLNDIFEAQKIEWHE**AAA

### Vti1 (126-190)

GSMLDRSTQRLKASQALAAETEAIGASMLAQLQQQREVIANTTRILYESEGYVDRSIKSLKGIARRM

### Vam7 (308-371)

GSQKLDEQEEYVKDIGVHVRRLRHLGTEIYNAIEQSKDDLDTLDQGLTRLGNGLDKAKALEKKVSGR

### DNA handle preparation

The DNA handle used in our single-molecule experiments is 2,260 bp in length and contains a thiol group (-SH) at one end and two digoxigenin moieties at the other end. The DNA handle was generated by PCR and purified using a PCR purification kit (Qiagen). Both labels were added to the 5′ ends of the PCR primers during synthesis.

### Protein purification

The coding sequences for rat or human syntaxin-1A, VAMP2, Munc18-1, and syntaxin-4 were cloned into pET-SUMO (Invitrogen), which introduced a His_6_-SUMO tag at the N-termini of the proteins. The coding sequences for rat SNAP-25B and SNAP-23 were cloned into pET-15b (Novagen), which introduced a His_6_ tag at the N-terminus of the protein. The coding sequence for rat Munc18-3 was cloned into pET-15a (Novagen) and codon-optimized for protein expression in bacteria (Morey et al., 2017). The plasmids were transformed into *Escherichia coli* BL21 (DE3) cells (Agilent Technologies), which were then grown in LB media supplemented with the appropriate antibiotics at 37°C until the OD at 600 nm reached 0.6–0.8. The cells were induced with 1 mM IPTG at 37°C for 5 hr. Variants of syntaxin-1A, VAMP2, SNAP-25B and Munc18-1 were prepared using standard PCR-based site-directed mutagenesis (Qiagen).

The neuronal SNARE proteins and Munc18-1 were purified using His-tag affinity purification, as previously described (Gao et al., 2012; Ma et al., 2015). Briefly, the cells were disrupted in HEPES buffer (25 mM HEPES, 400 mM KCl, 10% glycerol, 0.5 mM TCEP, pH 7.7) containing 10 mM imidazole and one tablet of EDTA free protease inhibitor cocktail (cOmplete, Roche). Cell lysates were cleared by ultracentrifugation. The resulting supernatant was mixed with Ni-NTA resin overnight, after which the resin was washed successively with HEPES buffer containing 20, 40, and 60 mM imidazole. SNAP-25B, VAMP2 and Munc18-1 were eluted in HEPES buffer containing 300 mM imidazole. Syntaxin-1A was eluted in biotinylation buffer (25 mM HEPES, 200 mM potassium glutamate, 300 mM imidazole, pH 7.7) for future biotinylation (see below). For VAMP2 and Munc18-1, the His_6_-SUMO tags were cleaved by SUMO proteases at 4*°*C *overnight. The cleaved tags were removed by binding to Ni-NTA resin followed by centrifugation.*

The *Chaetomium thermophilum* vacuolar SNARE motifs and Vps33 were purified using a previously described protocol with minor modifications (Baker et al., 2015). The *C. thermophilum* SNARE motifs were cloned into a modified pQLinkH vector, resulting in an N-terminal His_7_-MBP-tag. The plasmids were transformed into *E. coli* C43 (DE3) cells (Lucigen), which were grown in LB media supplemented with ampicillin at 37°C until the OD at 600 nm reached ~0.6. The cells were induced with 0.5 nm IPTG at 30°C for 4 hr and disrupted in lysis buffer (20 mM HEPES, pH 8.0, 350 mM NaCl, 10 mM β-mercaptoethanol and 1 mM PMSF) supplemented with 40 mM imidazole. The lysate was cleared by centrifugation at 17,000 g for 30 min. The His_7_-MBP-tagged SNARE domains and His_7_-tagged Vps33 were purified by binding to Ni-NTA resin for several hours, followed by three washes with lysis buffer supplemented with 40 mM imidazole, and elution in lysis buffer supplemented with 300 mM imidazole. For Vps33, the protein was concentrated, followed by size exclusion chromatography on a S200 column equilibrated with gel filtration buffer (20 mM Tris pH 8.0, 250 mM NaCl, 5% glycerol and 0.5 mM TCEP). For the SNARE domains, the His_7_-MBP tag was removed by incubation with TEV protease with a protein:protease ratio of 20:1 for 3 hr at room temperature. The sample was pre-cleared by running on a gravity flow amylose column and concentrated, followed by size exclusion chromatography on a S75 column equilibrated with gel filtration buffer. Residual His_7_-MBP was removed using a gravity flow amylose column.

After purification, Qa-SNAREs (syntaxin-1A and Vam3) were biotinylated at the Avi-tag in the presence of 50 µg/mL BirA, 50 mM bicine buffer, pH 8.3, 10 mM ATP, 10 mM magnesium acetate, and 50 μM d-biotin (Avidity) at 4°C overnight (Gao et al., 2012; Jiao et al., 2017).

### SNARE complex formation

To form synaptic SNARE complexes, syntaxin-1A, SNAP-25B, and VAMP2 were mixed in a molar ratio of 0.8:1:1.2 and incubated at 4°C overnight in the HEPES buffer (pH 7.7) with 2 mM TCEP. The SNARE complexes were purified using the His-tag on SNAP-25B (Gao et al., 2012). The quality of the purified neuronal SNARE complex was confirmed by its SDS-resistance in denaturing gel electrophoresis. To form *C. thermophilum* vacuolar SNARE complexes, the His-MBP-tagged Nyv1, Vam3, Vti1 and Vam7, 250 nmol each, were mixed and incubated overnight at 4°C. The complexes were separated from unbound SNAREs by size exclusion chromatography. The His-MBP-tags were cleaved from the SNARE domains using TEV protease and removed by binding to amylose resin. The vacuolar SNARE complex was stored in 20 mM Tris, pH 8.0, 250 mM NaCl, 5% glycerol, 0.5 mM TCEP.

### Crosslinking

After the SNARE complexes were formed, we crosslinked the R and Qa-SNAREs at the N-termini of their SNARE motifs and the R-SNARE C-terminus and the 2,260 bp-DNA handle. To this end, both SNARE complexes and DNA handles were treated with 2 mM TCEP for 1 hr at room temperature, after which Bio-Spin six columns (Bio-Rad) were used to change the buffer to crosslinking buffer A (100 mM phosphate buffer, 500 mM NaCl, pH 5.8) for DNA handles or crosslinking buffer B (100 mM phosphate buffer, 500 mM NaCl, pH 8.5) for SNARE complexes. Next, DNA handles were incubated with 1 mM 2,2'-dithiodipyridine disulfide (DTDP) for 1 hr at room temperature to activate the thiol group for the following crosslinking reaction. After incubation, the DNA handle was purified using a PCR purification kit and eluted in crosslinking buffer B to remove excess DTDP. Finally, the SNARE complexes were mixed with the DTDP-treated DNA handles in a 50:1 molar ratio in crosslinking buffer B and incubated at room temperature overnight, as previously described (Gao et al., 2012).

### Single-molecule manipulation experiments

All pulling experiments were performed using dual-trap high-resolution optical tweezers as previously described (Gao et al., 2012; Ma et al., 2019; Ma et al., 2015). Briefly, an aliquot of the crosslinked protein-DNA mixture containing 10–100 ng DNA was mixed with 10 μL 2.1 μm diameter anti-digoxigenin antibody coated polystyrene beads (Spherotech) and incubated at room temperature for 15 min. Then the anti-digoxigenin coated beads and 2 μL 1.7 μm diameter streptavidin-coated beads (Spherotech) were diluted in 1 mL PBS buffer (137 mM NaCl, 2.7 mM KCl, 8.1 mM Na_2_HPO_4_, 1.8 mM KH_2_PO_4_, pH 7.4). Subsequently, the bead solutions were separately injected into the top and bottom channels of a homemade microfluidic chamber as described below. The central channel contained PBS buffer with an oxygen scavenging system comprising 400 mg/mL glucose (Sigma-Aldrich), 0.02 unit/mL glucose oxidase (Sigma-Aldrich), and 0.06 unit/mL catalase (Sigma-Aldrich). A single anti-digoxigenin-coated bead was trapped and brought close to a single streptavidin-coated bead held in another optical trap to form a single SNARE-DNA tether between the two beads.

A single SNARE protein (Qa), SNARE conjugate (Qa-R), SNARE complex, or SNARE/SM complex (collectively called the protein complex below) was pulled or relaxed by moving one of the optical traps at a speed of 10 nm/s. In a typical single-molecule manipulation experiment, a single protein complex was first pulled to a high force to completely disassemble the complex, yielding information on the stability and structure of the complex. Then the complex was relaxed to observe its possible refolding or re-assembly. To better observe the assembly of the template complex or the SNARE complex, during relaxation the protein complex was often held at constant trap separations in a force range of 2–8 pN for various times. The formation probability of the complex generally increased as the waiting time increased. Therefore, the formation probability of the template complex or the SNARE four-helix bundle reported in the main text, including Table 1, was determined with a maximum waiting time of 1 min.

### Dual-trap high-resolution optical tweezers

The optical tweezers used in our experiments are home-built and described in detail elsewhere (Gao et al., 2012; Ma et al., 2015; Sirinakis et al., 2012). Briefly, the tweezers are assembled on an optical table located in an acoustically isolated, temperature- and air-flow-controlled room. A 1064 nm laser beam from a 4 W Nd:YVO4 diode pumped solid state laser (Spectr-Physics, CA) is expanded by a telescope by about five fold, and split by a polarizing beam splitter (PBS) into two orthogonally polarized laser beams. The two beams are reflected by two mirrors and combined by another PBS. One of the mirrors is mounted on a nano-positioning stage that can tip/tilt in two axes with high resolution (Mad City Labs, WI). The combined beams are further expanded by about twofold and collimated by another telescope, and focused by a water immersion 60X objective with a numerical aperture of 1.2 (Olympus, PA), forming two optical traps in a central channel of the microfluidic chamber. One of the optical traps can be moved in the sample plane with sub-angstrom resolution via the nano-positioning stage. The flow cell is formed between two coverslips sandwiched by Parafilm cut into three parallel channels. The top and bottom channels are connected to the central channel by glass tubing. The outgoing laser beams are collected and collimated by an identical objective, split again by a PBS, and projected onto two position-sensitive detectors (Pacific Silicon Sensor, CA), which detect displacements of the two beads in optical traps through back-focal-plane interferometry. The optical tweezers are calibrated before each single-molecule experiment by measuring the Brownian motion of the trapped beads, which yields the power-spectrum density distributions of bead displacements. The force constants of optical traps are determined by fitting the measured power-spectrum density distributions with a Lorentzian function.

### Circular dichroism (CD) spectra of Munc18-1

CD spectra of WT and mutant Munc18-1 proteins were measured in 20 mM phosphate buffer using an Applied Photophysics Chirascan equipped with a 2 mm quartz cell. The readings were made at 1 nm intervals, and each data point represents an average of 6 scans at a speed of 120 nm/min over the wavelength range of 190 to 250 nm.

### Derivations of protein unfolding energy and folding and unfolding rates from force-dependent measurements

Our methods of data analysis and the relevant Matlab codes are described in detail elsewhere (Gao et al., 2012; Rebane et al., 2016; Zhang et al., 2016a). Briefly, the extension-time trajectories obtained at constant trap separations or mean forces were first analyzed by two-state hidden-Markov modeling (McKinney et al., 2006; Zhang et al., 2016a), which revealed the idealized state transitions, extension changes, unfolding probabilities, and folding and unfolding rates. The MATLAB codes for hidden-Markov modeling can be found in Source code file 1. These measurements were used to derive the folding intermediates and their associated energy and kinetics.

We quantified the structural change of a single protein based on the measured force and extensions (Rebane et al., 2016). The control parameter of our pulling experiment is the separation between two optical traps (D). Given the trap separation, the extension (X) and tension (F) of the protein-DNA tether are calculated as

(2) $$X=D-x_{1}-x_{2}$$

(3) $$F=\left(F_{1}+F_{2}\right)/2,$$

respectively, where $x_{1}$ and $x_{2}$are displacements of the two beads in optical traps, and $F_{1}$ and $F_{2}$ are the corresponding forces applied to the beads. Both bead displacement x and the force Fare derived from voltage outputs of the position-sensitive detectors after proper calibrations (Sirinakis et al., 2012). In Equation 2, we have defined a default relative trap separation by neglecting the contribution of constant bead diameters. It is this relative trap separation that is shown in Figure 2—figure supplement 2. As a protein *m*olecule unfolds, its extension $\left(x_{m}\right)$ increases, which leads to retraction of both beads in their optical traps and the accompanying decrease in tension (Figure 2—figure supplement 2). Thus, during protein folding and unfolding transitions, the tether tension changes in an out-of-phase manner with respect to the tether extension, and thus is state-dependent. In the constant trap separation, the mean force is defined as the mean of the average forces associated with the folded and unfolded states (Rebane et al., 2016).

We modeled the unfolded peptide and the DNA handle by a worm-like chain model (Marko and Siggia, 1995). Based on this model, the stretching force F and the entropic energy E of a semi-flexible polymer chain are related to its extension x, contour length L, and persistence length P by the follow formulae

$$F=\frac{k_{B}T}{P}\left[\frac{1}{4{\left(1-\frac{x}{L}\right)}^{2}}+\frac{x}{L}-\frac{1}{4}\right]$$

(5) $$E=\frac{k_{B}T}{P}\frac{L}{4\left(1-\frac{x}{L}\right)}\left[3{\left(\frac{x}{L}\right)}^{2}-2{\left(\frac{x}{L}\right)}^{3}\right],$$

respectively. We adopted a persistence length 40 nm for DNA and 0.6 nm for the unfolded polypeptide (Gao et al., 2012; Ma et al., 2015; Rebane et al., 2016). Because the DNA extension ($x_{DNA}$) is known given a force or trap separation via Equation 4, the extension of the protein can be calculated as $x_{m}=X-x_{DNA}$. The protein extension generally comprises the extensions of the unfolded *p*olypeptide portion ($x_{p}$) and the folded portion (H) of the protein if any, or $x_{m}=x_{p}+H.$ The former can be again calculated by Equation 4, given the contour length of the unfolded polypeptide ($L_{p}$), whereas the latter can be treated as a force-independent constant, or a hard core of the protein (Rebane et al., 2016). Here, the size of the hard core is determined from the two pulling sites on the folded protein portion, which changes with the protein state. Thus, the contour length of the unfolded polypeptide and the size of the folded protein portion are correlated and can be determined based on a structural model for protein transitions. To derive the structure of the template complex, we assumed a change in the hard core size of 3 nm for the transition from the partially closed syntaxin to the folded template complex, as determined from the structure-based model (Baker et al., 2015). Consequently, we could determine the contour length of the polypeptide chain in the Qa-R conjugate that is either free or bound by Munc18-1. The number of amino acids in a polypeptide is its contour length divided by the contour length per amino acid, which is chosen to be 0.365 nm (Gao et al., 2012; Rebane et al., 2016). The number of amino acids in the completely unfolded SNARE state 5 is known (Figure 2—figure supplement 1), which helps derive the structure of the template complex based on the extension change during the template complex transition. We determined that 87 (±2, S.D.) amino acids are sequestered in the folded template complex, including the N-terminal loop formed between syntaxin-1 and VAMP2 due to crosslinking. Based on our construct design (Figure 2—figure supplement 1), this length is consistent with the structure of the predicted template complex (Figure 1A).

Similarly, we modeled the total free energy of the whole dumb-bell system in optical traps, or

(6) $$G=\frac{F^{2}}{2k_{traps}}+E_{DNA}+E_{p}+V,$$

where the first term represents the potential energy of the two beads in optical traps with $k_{traps}=k_{1}k_{2}/\left(k_{1}+k_{2}\right)$ the effective force constant of the two traps, the second and third terms are entropic energies of the DNA handle and the unfolded polypeptide, respectively, calculated by Equation 5, and the last term is the free energy of the protein at zero force. Based on the Boltzmann distribution, the protein unfolding energy ΔV can be determined by fitting the measured unfolding probability using Equation 6. Equation 6 can be similarly applied to the transition state of protein folding (Rebane et al., 2016). With Kramers’ rate equation, the folding and unfolding rates are calculated. By fitting the calculated rates to the measured rates, we derive the energy and conformation of the transition state, which also yield the folding and unfolding rates at zero force. Complete data sets from individual molecules are separately fit and the unfolding energies and transition rates, typically averaged over more than three different molecules, are reported (Table 1). The average folding rates and unfolding rates of the WT and mutant template complexes fall in the ranges of 17–568 s^−1^ and 0.1–10 s^−1^, respectively, with a standard error typically close to the corresponding average rate for each template complex.

### Estimation of the affinity between VAMP2 and Munc18-bound syntaxin in the absence of crosslinking

The N-terminal crosslinking between Qa- and R-SNAREs used in our assay is crucial for us to observe and characterize the template complex. The crosslinking destabilizes the closed syntaxin, thereby bypassing the requirement for Munc13-1; mitigates SNARE misassembly, for example, formation of various anti-parallel SNARE bundles (Lai et al., 2017); and avoids nonspecific VAMP2-Munc18-1 interactions (Sitarska et al., 2017). Therefore, the crosslinking simplifies our experimental design and data interpretation. Three lines of evidence suggest that the crosslinking does not compromise the major conclusions derived from our assay. First, the stability (5.2 ± 0.1 k_B_T vs 4.8 ± 0.3 k_B_T) and lifetime (1.4 s vs 0.8 s) of the template complex do not significantly depend upon the crosslinking site used in our assay (at R198C vs I187C, Figure 2—figure supplements 1 and 7). This observation suggests that the crosslinking does not alter the structure of the template complex and is likely located at a disordered region. The approximate independence of the folding energy and lifetime on the crosslinking site has been extensively tested in other systems, such as DNA hairpins (Woodside et al., 2006) and protein coiled coils (Jiao et al., 2015). Second, the derived template model recapitulates many distinct features of the fusion machinery, including its dependence upon NRD, phosphorylation, and various mutations. Finally, the crosslinking increases the local SNARE concentration around Munc18-1, which mimics the environment of SNARE assembly and membrane fusion in vivo due to vesicle tethering and SNARE recruitment. For example, Munc13-1 essentially crosslinks both syntaxin and VAMP2 by simultaneously binding the two (Sitarska et al., 2017; Wang et al., 2017) (Figure 1).

The effective concentration due to the crosslinking can be quantified, which is used to estimate the binding affinity between VAMP2 and partially closed syntaxin bound by Munc18-1 in the absence of crosslinking (Zhang et al., 2016b). To derive the local concentration of the crosslinked VAMP2 (at R198C) around the partially closed syntaxin bound by Munc18-1, we made three assumptions: 1) the partially closed syntaxin bound by Munc18-1 has a conformation similar to the conformation seen in the crystal structure of the Vps33:Vam3 complex (Baker et al., 2015); 2) The kinetics of VAMP2 binding and unbinding are dominated by Phe 77 insertion into and detachment from the F-binding pocket on the Munc18-1 surface, respectively, as is supported by our data; and 3) the unfolded SNARE polypeptides are described by a Gaussian chain model. Thus, VAMP2 binding to the partially closed syntaxin bound by Munc18-1 can be modeled by Phe 77 binding to the F-binding pocket while Phe 77 is tethered to the −7 layer of Vam3 (i.e. the N-terminus of the main Vam3 helix in the Vps33:Vam3 structure, PDB code 5BUZ) via a polypeptide linker. The linker length is 54 amino acids (or *L* = 19.7 nm in terms of the contour length) for the Qa-R conjugate crosslinked at R198C. The distance between the F-binding pocket and the tethering point (*R*) is measured to be 6.34 nm. Therefore, the effective concentration *c* of the tethered Phe 77 around its binding pocket is calculated as *c* = 3.7 × 10^−4^ M, using the following formula

(7) $$c=\frac{1}{N_{A}}{\left(\frac{3}{4\pi PL}\right)}^{\frac{3}{2}}exp\left(-\frac{3R^{2}}{4PL}\right),$$

where $N_{A}$ is Avogadro’s number and p=0.6 nm is the persistence length of the polypeptide. Due to coupled binding and folding, the folding and unfolding rates of the template complex we measured should be equal to the binding and dissociation rates estimated here. Therefore, the folding rate *k_f_* = *k_on_* × c, where *k_on_* is the bimolecular rate constant for VAMP2 binding to the partially-closed syntaxin. Using our measured folding and unfolding rates for the R198C conjugate, we calculated the VAMP2 binding rate constant as *k_on_* = 3.5 × 10^5^ s^−1^M^−1^, energy as 13.1 k_B_T, or a VAMP2 dissociation constant as 2 µM. Supposing that VAMP2 binding to the fully closed syntaxin requires an energy gain of 4.6 k_B_T compared to the partially closed syntaxin (corresponding to the energy difference between the two syntaxin states), the VAMP2 binding affinity to the fully closed syntaxin is estimated to be 200 µM. This estimation justifies a weak template complex that is difficult to detect in vitro using conventional experimental approaches, but can be observed using our single-molecule approach in combination with N-terminal SNARE crosslinking.

The calculated local concentration of VAMP2 Phe 77 around the F-binding pocket for the Qa-R conjugate crosslinked at I187C (*c* = 4.3 × 10^−4^ M) is slightly higher than that for the R198C conjugate (*c* = 3.7 × 10^−4^ M). Correspondingly, the unfolding energy of the template complex for the I187C conjugate is expected to be slightly higher by 0.15 k_B_T, given their equal unbinding rate based on our model. Therefore, our simple molecular model for the template complex transition recapitulates our experimental observations that the stability and lifetime of the template complex is insensitive to the crosslinking site used in our assay.

### Data and software availability

Data and software can be found in the Source data and the Source code file, respectively.

## Additional files

Transparent reporting form

### Data availability

All data generated or analyzed during this study are included in the manuscript and supporting files. Source data files have been provided for Figures 2-10.
